# Patterns of Population Structure and Introgression Among Recently Differentiated *Drosophila melanogaster* Populations

**DOI:** 10.1093/molbev/msac223

**Published:** 2022-10-17

**Authors:** Jenn M Coughlan, Andrius J Dagilis, Antonio Serrato-Capuchina, Hope Elias, David Peede, Kristin Isbell, Dean M Castillo, Brandon S Cooper, Daniel R Matute

**Affiliations:** Biology Department, University of North Carolina, Chapel Hill, NC, USA; Department of Ecology and Evolutionary Biology, Yale University, New Haven, CT, USA; Biology Department, University of North Carolina, Chapel Hill, NC, USA; Arnold Arboretum, Harvard University, Cambridge, MA, USA; Biology Department, University of North Carolina, Chapel Hill, NC, USA; Department of Ecology and Evolutionary Biology, Brown University, Providence, RI, USA; Center for Computational Molecular Biology, Brown University, Providence, RI, USA; Biology Department, University of North Carolina, Chapel Hill, NC, USA; Institute of Agriculture and Natural Resources, University of Nebraska-Lincoln, Lincoln, NE, USA; Division of Biological Sciences, University of Montana, Missoula, MT, USA; Biology Department, University of North Carolina, Chapel Hill, NC, USA

**Keywords:** demography, genetic differentiation, genetic incompatibility, gene flow, reproductive isolation

## Abstract

Despite a century of genetic analysis, the evolutionary processes that have generated the patterns of exceptional genetic and phenotypic variation in the model organism *Drosophila melanogaster* remains poorly understood. In particular, how genetic variation is partitioned within its putative ancestral range in Southern Africa remains unresolved. Here, we study patterns of population genetic structure, admixture, and the spatial structuring of candidate incompatibility alleles across a global sample, including 223 new accessions, predominantly from remote regions in Southern Africa. We identify nine major ancestries, six that primarily occur in Africa and one that has not been previously described. We find evidence for both contemporary and historical admixture between ancestries, with admixture rates varying both within and between continents. For example, while previous work has highlighted an admixture zone between broadly defined African and European ancestries in the Caribbean and southeastern USA, we identify West African ancestry as the most likely African contributor. Moreover, loci showing the strongest signal of introgression between West Africa and the Caribbean/southeastern USA include several genes relating to neurological development and male courtship behavior, in line with previous work showing shared mating behaviors between these regions. Finally, while we hypothesized that potential incompatibility loci may contribute to population genetic structure across the range of *D. melanogaster;* these loci are, on average, not highly differentiated between ancestries. This work contributes to our understanding of the evolutionary history of a key model system, and provides insight into the partitioning of diversity across its range.

## Introduction

*Drosophila melanogaster* remains one of the most powerful genetic systems to understand the molecular underpinnings of phenotypic and fitness variation since its development in the early 20th century (Morgan 1910, 1911; Green 2010). While *D. melanogaster* is commonly associated with human settlements, it likely originated in the African Mopane and Miombo forests, where extant wild populations still breed on marula fruits far from human settlements (Mansourian et al. 2018; Sprengelmeyer et al. 2020). The transition to commensalism within Africa may have then allowed for range and dietary expansion approximately 10,000–13,000 years ago, before a rapid global expansion shortly thereafter (proposed by David and Capy (1988), Lachaise et al. (1988), Baudry et al. (2004), Thornton and Andolfatto (2006), Singh et al. (2007), Stephan and Li (2007), more recently examined by Duchen et al. (2013), Adrion et al. (2015), Mansourian et al. (2018), Arguello et al. (2019), Sprengelmeyer et al. (2020). Given the importance of model systems, like *D. melanogaster* and closely related *Drosophila* species, to our understanding of the genetic basis of morphological (e.g., Kopp et al. 2000; McGregor et al. 2007), physiological (e.g., Montooth et al. 2003), and behavioral (e.g., Ding et al. 2016; York et al. 2021) traits, as well as our understanding of different evolutionary processes in both natural and experimental contexts (Harshman and Hoffmann 2000; Markow 2015; White et al. 2020), it remains critical to understand how genetic variation is partitioned in the ancestral range of model organisms.

Significant population genetic structure has been described for *D. melanogaster* outside of Africa (Grenier et al. 2015; Arguello et al. 2019; Kapun et al. 2020; Machado et al. 2021; Yue et al. 2021; Kapun et al. 2022), between African and non-African populations of *D. melanogaster* (Begun and Aquadro 1993; Bénassi and Veuille 1995; Nunes et al. 2008; Duchen et al. 2013; Kapopoulou, Kapun, et al. 2018a), and more recently within Africa (David and Capy 1988; Vouidibio et al. 1989; Dieringer et al. 2005; Pool and Aquadro 2006; Pool et al. 2012; Lack et al. 2015; Kern and Hey 2017; Kapopoulou, Pfeifer, et al. 2018b; Sprengelmeyer et al. 2020). Early multilocus or isozyme surveys found limited to modest structure within Africa (Bénassi and Veuille 1995; Dieringer et al. 2005; Schlötterer et al. 2006), supporting distinct West and East African clades (Pool and Aquadro 2006). More recent efforts suggest that extant wild populations that may closely resemble the ancestor to modern *D. melanogaster* exist as isolated, genetically unique clades within the putative ancestral range (Pool et al. 2012; Lack et al. 2015; Mansourian et al. 2018; Sprengelmeyer et al. 2020). Despite these efforts, genetic differentiation within the ancestral range of Southern Africa is largely still unresolved, in part due to a lack of sampling from more remote areas in the Mopane and Miombo forests. Moreover, patterns of gene flow between African ancestries are largely unexplored (though see Kern and Hey (2017), Medina et al. (2018)). Understanding patterns of genetic structure and connectivity within the ancestral range are essential to unraveling the evolutionary history of *D. melanogaster*.

Human-aided migration following the transition to human commensalism is thought to have contributed to range expansion in *D. melanogaster* both within Africa (Adrion et al. 2015; Mansourian et al. 2018; Sprengelmeyer et al. 2020), and globally via a single out of Africa event (Baudry et al. 2004; Grenier et al. 2015; Arguello et al. 2019; Sprengelmeyer et al. 2020), characterized by multiple bottlenecks (Haddrill et al. 2005; Thornton and Andolfatto 2006). After this expansion, multiple historical events created opportunities for human-mediated admixture between genetically distinct lineages of *D. melanogaster.* Identifying genetic lineages of *D. melanogaster*—both within and outside of Africa—is crucial to better understand admixture events. For example, the transatlantic movement of enslaved peoples roughly 400 years ago has been hypothesized to have produced a secondary contact zone between African and non-African populations of *D. melanogaster* in the southeastern USA and the Caribbean (Caracristi and Schlötterer 2003; Yukilevich and True 2008; Duchen et al. 2013; Kao et al. 2015; Bergland et al. 2016), but it is unknown whether different genetic lineages within Africa contributed to this admixture event. Moreover, non-African lineages have also contributed to the diversity exemplified in modern African populations via back migration. In particular, the opening of western commercial routes and the “Scramble for Africa” potentially facilitated hybridization between local African populations of *D. melanogaster* and invading non-African *D. melanogaster* individuals (Caracristi and Schlötterer 2003; Medina et al. 2018). Indeed, the extent of non-African ancestry in Africa is widely variable between populations (Lack et al. 2015), with some evidence for more pronounced signatures of admixture in urban populations (Capy et al. 2000; Kauer et al. 2003).

In addition to genome-wide patterns of population genetic structure, the structuring of particular genetic variants can impact fitness variation within and among populations. Here, we focus on just two types of loci which are common and have some established fitness effects in *D. melanogaster*: epistatically interacting loci that contribute to fitness variation and chromosomal inversions. First, different combinations of epistatically interacting loci can create substantial fitness variation in *D. melanogaster* (Corbett-Detig et al. 2013; Pool 2015). Similar to hybrid incompatibilities, these epistatic interactions can produce low fitness individuals within a species (Sweigart et al. 2007; Cutter 2012; Corbett-Detig et al. 2013; Pool 2015; Zuellig and Sweigart 2018). It has been hypothesized that such candidate incompatibilities may be differentiated between certain populations of *D. melanogaster* (Pool 2015), but we lack an understanding of if and how these loci are structured throughout its range. Second, structural genomic changes, such as chromosomal inversions, can also play an important role in both adaptive processes as well as population genetic inference (reviewed in Wellenreuther and Bernatchez (2018), Faria et al. (2019)). In *D. melanogaster*, chromosomal inversions have long been associated with environmental adaptation (Kapun et al. 2014, 2016a, 2016b; Durmaz et al. 2018; Kapun and Flatt 2019; McBroome et al. 2020; Sprengelmeyer et al. 2020; Machado et al. 2021). Despite their potential importance for adaptation, the frequencies of common inversions have not been well described in the ancestral range of *D. melanogaster,* predominantly due to a lack of sampling in remote regions of Southern Africa (though see [Sprengelmeyer et al. 2020]). Regardless of their adaptive value, chromosomal inversions can also distort patterns of diversity and divergence (Corbett-Detig and Hartl 2012; Pool et al. 2012), and it is therefore important to take into account known inversions when interpreting population genomic data. Determining how candidate incompatibility loci and chromosomal inversions correspond with population structure and patterns of gene flow can contribute to our understanding of the factors that shape genetic diversity in natural populations.

Here we address the extent of genetic differentiation and gene flow across a global sample of *D. melanogaster*, with particular focus on population differentiation within the presumed ancestral range in Southern Africa. We also implement a new linear discriminant analysis (LDA) approach to quantify the frequency of nine common inversions across Southern Africa. Lastly, we describe the geographic distribution of previously identified candidate incompatibility alleles and their contributions to admixture and population structure. Our results help to clarify the demographic history of *D. melanogaster* and provide some insights into the persistence of genetically unique clades within *D. melanogaster*.

## Results

### Diversity, Divergence, and Evolutionary Relationships Among Populations of *Drosophila melanogaster*

To understand the global distribution of diversity and population structure of *D. melanogaster,* we combined whole genome resequence data from 803 lines of *D. melanogaster* sampled globally, including 223 newly sequenced genomes, 190 of which originate from previously unsampled or undersampled rural locales within the presumed ancestral range in Southern Africa.

**Fig. 1.**
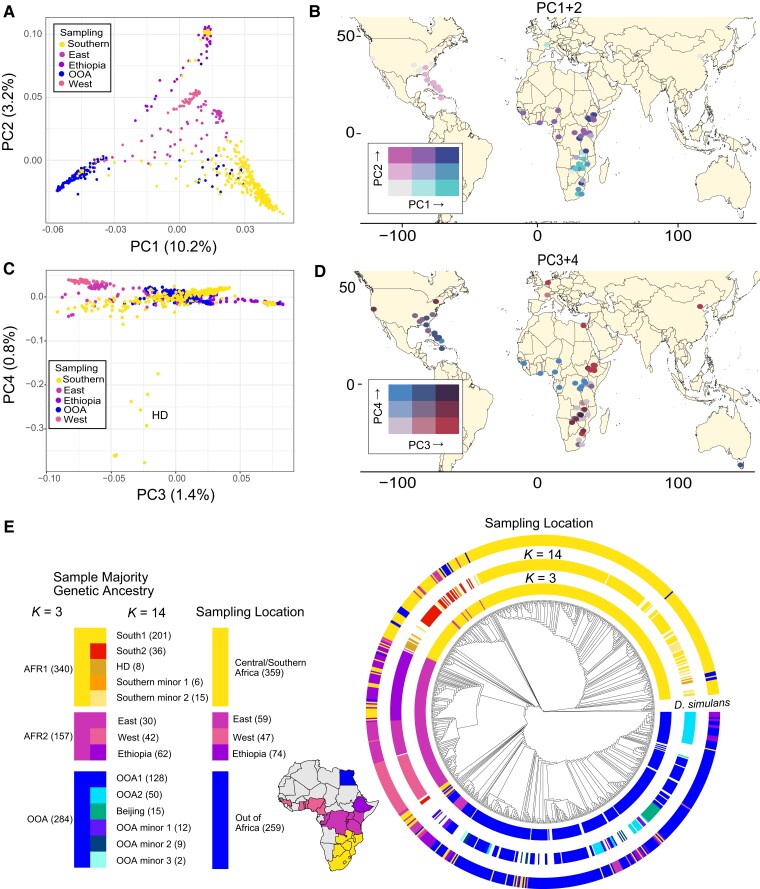
The structuring of genetic diversity across a broad sampling of *Drosophila melanogaster*. (*A* and *B*) PCs 1 and 2 among all *D. melanogaster* samples define three major clades. PC1 distinguishes Southern African from Out of Africa samples, while PC2 distinguishes Central African samples from all others. (*C* and *D*) PCs 3 and 4 separate samples along a latitudinal gradient and identify a distinct lineage in Southern Africa, respectively. PC3 increases with latitude, both in and out of Africa, while PC4 reveals a cryptic, monophyletic clade which we refer to as HD. (*E*) Phylogenetic relationship and geographic sampling of 811 genomes from a global distribution of *D. melanogaster*; ASTRAL consensus tree of 100 kb windowed ML trees of collinear regions of the genome among the samples. Inner ring labels samples based on majority ancestry when *K* = 3, middle for *K* = 14 and outer indicates sampling locale, with a map of sampling locale labels within Africa. Tree is rooted using samples of *D. simulans*.

A principal component (PC) analysis based on collinear regions of the genome revealed that genetic variation within *D. melanogaster* is mainly structured between flies from Southern Africa and Out of Africa (OOA), as reflected by PC1 (which explains 10.2% of the variation). While somewhat intermediate along PC1, flies collected from East and West Africa, are much more distinct from OOA and Southern Africa along PC2 (which explains 3.2% of the total variation; fig. 1). Flies collected from Ethiopia exhibited the most extreme values of PC2 and are largely distinct from flies collected elsewhere in East Africa (fig. 1). When these PCs are projected onto a map, the global structuring of variation within *D. melanogaster* is striking, with distinct shifts in ancestry between Southern and Central Africa, and between the African continent and Europe and North America. Flies collected from the southeast USA and the Caribbean show similarities with flies from Central Africa along PCs 2 and 3, resulting in subtle differences in ancestries within North America.

To explicitly compare diversity within and divergence among ancestry types, we used *NGSAdmix* (Skotte et al. 2013) and *PCAngsd* (Meisner and Albrechtsen 2018) to infer the number of distinct ancestry types (*K*) and assign ancestry to each individual. These methods varied significantly in the number of inferred ancestry types, with *NGSAdmix* inferring *K* = 3 as most likely (supplementary fig. S1 and S2, Supplementary Material online), while *PCAngsd* inferred *K* = 14 distinct ancestry types (based on the number of significant PCs + 1; supplementary fig. S1 and S2, Supplementary Material online). Differences between these methods largely result from *PCAngsd* defining substructure within the 3 ancestries that *NGSAdmix* inferred. This includes defining both previously described ancestries (i.e., Beijing; [Grenier et al. 2015; Arguello et al. 2019]) and newly discovered structure (see below). When *K* = 3, we find that these ancestries largely correspond to samples from Southern Africa (AFR1), Central Africa (AFR2; i.e., East and West Africa), and all OOA lines. At *K* = 14 we identify eight ancestries that predominantly occur in Africa: five of which are most common in Southern Africa, and one that is most common in each of East Africa (excluding Ethiopia), West Africa, and Ethiopia. The OOA lines comprise three ancestries, one ancestry type that is more common in Europe, Egypt, and Tazmania, one that is more common in North America and the Caribbean, and one ancestry that is most common in Beijing (supplementary fig. S3, Supplementary Material online). The remaining three ancestries are present globally and occur predominantly as minor ancestries (i.e., ancestries that are relatively rare and most often occur at frequencies <50% in any given individual). Of these minor ancestries, we find that one occurs primarily in Africa (Global minor 1), and two are present globally (Global minor 2 and 3; fig. 1, supplementary fig. S3, Supplementary Material online). As all individuals showed some level of mixed ancestry, we used the majority ancestry present in each individual (i.e., the ancestry type >50%) to assign individuals to discrete ancestry groups. Some individuals (particularly at *K* = 14) did not have a single ancestry >50%, and so were excluded from further analyses.

Our *K*-means clustering and PC analyses largely agree with our consensus phylogeny of these lineages (fig. 1*A*). The genome-wide consensus phylogeny was based on individual ML phylogenies of 811 fly lines (five lines of *D. melanogaster* were excluded due to low read depth, and 13 *D*. *simulans* lines were included as an outgroup) in non-overlapping 100 kb windows across collinear regions of the genome. This phylogeny predominantly groups samples by geography and the ancestries we have identified (fig 1*A*). When using *K* = 3, each majority ancestry type is largely monophyletic (fig. 1*A*). An interesting exception to this pattern is a small group of individuals with a majority Central African ancestry (collected originally from Ethiopia, Rwanda, Kenya, and Uganda) that are sister to the OOA clade. When using *K* = 14 ancestries, monophyly of each ancestry is variable. Of these 14 ancestry types, nine are commonly the majority of ancestry (i.e., >50% of the ancestry within an individual). Of these nine majority ancestries, seven are largely monophyletic across the autosomal genome. These include three ancestries that are most common in or unique to flies collected in Southern Africa; one each for flies collected from West Africa, East Africa, and Ethiopia; and one ancestry group that is most common in flies collected from Beijing. The remaining two major ancestries are most common in OOA lines, with one being more common in flies collected from North America and the Caribbean and one being more common in flies collected from Europe, Tasmania, and Egypt (fig. 1). While the autosomal consensus tree suggests that these two OOA ancestries are paraphyletic, the X chromosome consensus tree suggests a monophyletic relationship (supplementary fig. S4, Supplementary Material online). We note that although the majority of these monophyletic clades and ancestry types are most frequent in a specific geographic area, there is some variation in whether an individual's sampling location matches their major ancestry type (fig. 1*A*, supplementary fig. S3, Supplementary Material online). These mismatches may hint at recent migration or admixture events (discussed below). Finally, the few individuals whose majority ancestry is one of the remaining five minor ancestries are placed throughout the tree. Individuals with a majority ancestry with one of the two minor African ancestries (South minor 1 and 2) are scattered throughout the broader South1 and South2 major ancestry types. These individuals are largely flies collected in Southern Africa, as well as four individuals originally collected in France. Individuals whose majority ancestry was one of the Global minor ancestries (i.e., Global minor 1, 2, and 3) were predominantly samples collected in Africa (including Egypt) that phylogenetically cluster with OOA individuals (fig. 1).

Using these two ancestry designations (*K* = 3 and nine major ancestries), we next sought to assess global differentiation, divergence, and diversity to further clarify evolutionary relationships and histories among ancestries. When using *K* = 3 ancestries, *F*_ST_ ranged between 0.076 and 0.161, with the lowest global *F*_ST_ found between AFR1 and AFR2 (*F*_ST_ = 0.076), and OOA being relatively more differentiated from both AFR2 and AFR1 (*F*_ST_ = 0.143 and 0.162, respectively; supplementary tables S2 and S8, Supplementary Material online). All pairwise comparisons of *D*_XY_ were very similar, with OOA and AFR2 exhibiting the lowest *D*_XY_ (supplementary table S2, Supplementary Material online). For the nine major ancestries the range of pairwise global *F*_ST_ was larger (0.046–0.367). Variation in *F*_ST_ was linked to both geographic distance and variation in within-species diversity. In general, geographically proximate ancestries were less differentiated and ancestries with lower diversity had higher levels of *F*_ST_ (supplementary table S2 and fig. S5, Supplementary Material online; in line with [Noor and Bennett 2009; Cruickshank and Hahn 2014]). *D*_XY_ was mostly low and was less variable among comparisons, although we find two notable exceptions (supplementary table S2, Supplementary Material online). First, the Southern African ancestry that is sister to all other ancestries (South1) showed elevated *D*_XY_ in every comparison (including with other ancestries from Southern Africa). Second, we find much reduced *D*_XY_ between all OOA lineages (supplementary table S2 and fig. S5, Supplementary Material online).

**Fig. 2.**
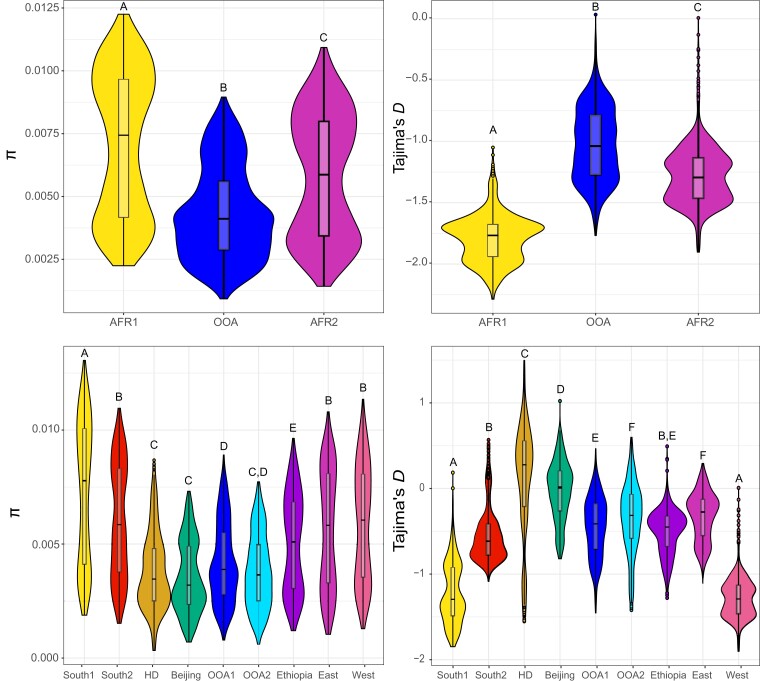
Genome-wide statistics for each genetic lineage. Average nucleotide diversity (*π*) and Tajima's *D* for each major ancestry type. Top: *K* = 3, the Southern African lineage (AFR1) shows both elevated *π* and reduced Tajima's *D*, with intermediate values of East/West/Ethiopia (AFR2) and lowest *π*/highest Tajima's *D* among the OOA lineage. Bottom: The nine major lineages identified with *K* = 14 largely replicate the relationships seen among the three lineages, with Southern African samples showing high diversity and low Tajima's *D*, while OOA samples show a diversity and Tajima's *D* consistent with their recent bottleneck and subsequent population expansion. A notable exception is the HD lineage in Southern Africa, which appears to have undergone a recent bottleneck.

To better infer how demographic histories varied among our ancestry types, as well as among chromosomes of the same ancestry types, we calculated the Site-Frequency Spectrum (SFS) and Tajima's *D* for each chromosome arm of each ancestry. We also calculated Tajima's *D* for synonymous and nonsynonymous sites across the genome for each ancestry to better understand how demographic and selective forces have shaped the distribution of allele frequencies. Ancestries varied in the extent of genetic variation and the distribution of allele frequencies (fig. 2). Under both *K* = 3 and the nine major ancestry classifications, the ancestries that exhibited higher diversity generally had an excess of rare variants, as demonstrated by both more negative values of Tajima's *D*, and a left-skewed SFS, indicative of a larger effective population size, a potential recent history of population expansion and purifying selection (fig. 2, supplementary fig. S6, Supplementary Material online). For both ancestry designations, individuals from Southern Africa generally had the highest diversity, while OOA individuals generally had the lowest, in line with (Duchen et al. 2013; Grenier et al. 2015; Arguello et al. 2019). For the nine major ancestries, the South1 ancestry type—which comprised mainly individuals from rural locales within Southern Africa and is sister to all other lineages in our phylogeny—had the highest diversity among all lineages (i.e., South2 and HD; fig. 1*A* and 2). South1 also showed higher diversity than almost all between-ancestry *D*_XY_ values (supplementary table S2, Supplementary Material online), suggesting that most diversity within *D. melanogaster* is merely a subset of the diversity within Southern Africa (in line with [Verspoor and Haddrill 2011; Pool et al. 2012; Arguello et al. 2019]). In contrast, South2 had diversity levels on par with East and West Africa (fig. 2), while the third ancestry from Southern Africa—which we name Harare Distinct (HD)—had the second lowest diversity of all ancestries and a shift in SFS to more intermediate variants (supplementary fig. S6, Supplementary Material online). HD is a monophyletic clade on both the autosomes and X chromosome (supplementary fig. S4, Supplementary Material online), and is distinct from all samples on PC4 (fig. 1*D*). Clades from OOA all showed low diversity, with Beijing individuals showing the lowest diversity (fig. 2). Similarly, all OOA clades exhibited more positive values of Tajima's *D* and a right-shifted SFS relative to other ancestries (fig. 2, supplementary fig. S6, Supplementary Material online). Differences in Tajima's *D* between chromosomes depended on the genetic lineage (based on an ANOVA with Type III SS: chromosome arm type × genetic lineage effect: *F* = 162.5, *df* = 8, *P* < 0.0001; supplementary fig. S7, Supplementary Material online). For five lineages, the X chromosome had lower values of Tajima's *D* (all three ancestries from Southern Africa, and East and West Africa), while Ethiopia and all three OOA ancestries showed the opposite pattern (fig. 2; supplementary fig. S7, Supplementary Material online). For all ancestries, nonsynonymous sites showed lower Tajima's *D* than synonymous sites, in line with the hypothesis that each ancestry has experienced some degree of purifying selection (supplementary fig. S20, Supplementary Material online). For the nine major ancestries, we note that South1 exhibited the largest difference in Tajima's *D* between synonymous and nonsynonymous sites, while HD exhibited the lowest (supplementary fig. S20, Supplementary Material online), suggesting that while South1 may have experienced the strong histories of purifying selection, HD has experienced a lower efficacy of purifying selection. Overall, these results suggest that demographic and/or selective history varies both between lineages and chromosome arms, with large differences even among closely related ancestries located in the same geographic locales (for instance, South1, South2 and HD or Ethiopia vs. the rest of East Africa).

### Patterns of Gene Flow Throughout the Range of *Drosophila melanogaster*

To better understand the sources and dynamics of gene flow across the range of *D. melanogaster*, we evaluated the extent of gene flow among distinct lineages within a global sampling of *D. melanogaster*. While we calculated Patterson's *D* statistics for all possible trios (65 unique trios in total), we focus on three potential cases of gene flow: (1) between Southern Africa and other African lineages, (2) between African and OOA lineages, and (3) the source(s) of African ancestry in the southeastern USA (as proposed by Yukilevich and True (2008), Duchen et al. (2013), Kao et al. (2015), Bergland et al. (2016).

Using only collinear regions of the genome, we find very few cases of significant introgression after Bonferroni correction for all trios (17/65 trios; supplementary table S3 and fig. S8, Supplementary Material online). Within Africa, we find evidence of gene flow among ancestries in Southern Africa, including between South1 and both HD and South2 (although not between South2 and HD; supplementary table S3 and fig. S8, Supplementary Material online). We also see weaker evidence of gene flow between each of East and West Africa and all three ancestries within Southern Africa, although these comparisons are largely not significant after Bonferroni correction (supplementary table S3 and fig. S8, Supplementary Material online). One exception to this trend is evidence for significant gene flow between West Africa and HD (supplementary table S3, fig. S8 and fig. S10, Supplementary Material online). We note that these shared signals of introgression are unlikely to represent multiple independent incidences of introgression, and more likely represent one or few bouts of introgression; either with the ancestral population that expanded into Central Africa or introgression with one Central African ancestry with subsequent migration among Central African ancestries. Additionally, these analyses were based on majority ancestry (rather than on collection locale), which may obscure more recent examples of gene flow when individuals from different locales phylogenetically cluster with another. We also excluded individuals with no major ancestry that may also represent more contemporary examples of gene flow. For instance, some individuals collected in Ethiopia cluster phylogenetically and on PCAs, and share major ancestries with Southern African individuals; and similarly, several lines collected in Southern Africa cluster with Ethiopian samples (fig. 1, supplementary fig. S4, Supplementary Material online). These samples may represent more contemporary examples of gene flow which would be undetectable using phylogenetically accurate *D* statistics. Lastly, we find evidence of greater gene flow between Ethiopia and West Africa than Ethiopia and the rest of East Africa, consistent with previous ancestry-based analyses (Lack et al. 2015; Medina et al. 2018). Overall, the patterns suggest both historical and more contemporary gene flow, sometimes between quite geographically disparate regions within Africa.

We next evaluated the extent of introgression between each OOA lineage and each African lineage. Again, we find very few trios with significant Patterson's *D* values after Bonferroni correction (8/33; supplementary table S3 and fig. S8, Supplementary Material online). While there is no evidence of gene flow between any Southern African ancestry and any OOA ancestry using *D* statistics (supplementary table S3 and fig. S8, Supplementary Material online), we note that multiple lines collected in France cluster phylogenetically with individuals from Southern Africa and carry major ancestries that are most common in Southern Africa (fig. 1*A*). Similarly, several lines collected in South Africa cluster with the OOA clade. As in above, these mismatches in clustering may represent more recent examples of admixture between ancestries, including evidence of back migration of OOA ancestries into South Africa (as has been suggested by Vouidibio et al. (1989), Capy et al. (2000), Caracristi and Schlötterer (2003), Kauer et al. (2003), Pool and Aquadro (2006), Pool et al. (2012), Lack et al. (2015), Medina et al. (2018), Svedberg et al. (2021)).

**Fig. 3.**
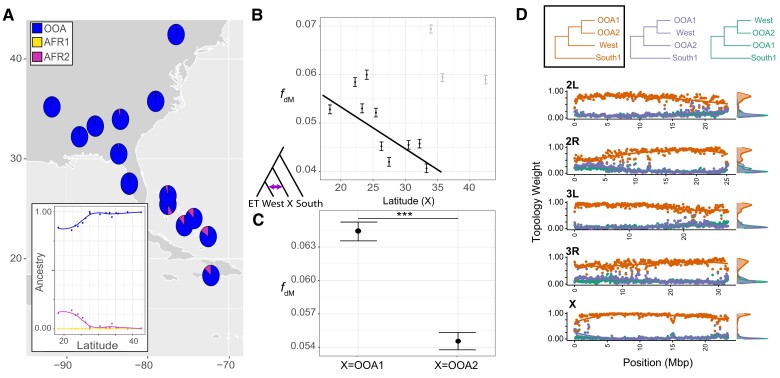
Patterns of gene flow between West Africa and different non-African lineages of *D. melanogaster*. (*A*) Latitudinal cline in Central African ancestry across the Caribbean and USA. No such cline exists for Southern African ancestry. Qualitatively similar results are found if using *K* = 14 (supplementary fig. S9, Supplementary Material online). (*B*) Median *f*_dM_ for multiple trios across a latitudinal cline, each calculated in 20 SNP windows for the following trios ([Ethiopia, West], X), where X denotes a collection site in the Caribbean or USA. A negative cline is found across the Caribbean and most southerly collections, but elevated levels of introgression are seen for Georgia, North Carolina, and Ithaca (denoted in gray). (*C*) A similar analyses to (*B*), but *f*_dM_ is calculated for all individuals belonging to either the OOA1 or OOA2 major ancestry, which correspond to lines collected from the Caribbean and southeastern USA, or Europe, North Africa, and Tazmania, respectively. (*D*) Weighted topologies calculated using *twisst* for West, OOA1 and OOA2. Although the vast majority of trees support a sister relationship between the two OOA ancestries, slightly more windows support a topology of ([West, OOA1], OOA2) than ([West, OOA2], OOA1), indicative of introgression.

In contrast to patterns in Southern Africa, we find that some Central African ancestries show significant introgression with several OOA ancestries. In particular, both West Africa and Ethiopia show evidence of introgression with both Beijing and OOA2 (supplementary table S3 and fig. S8, Supplementary Material online). We also see several lines where the sampling location is at odds with the majority ancestry the line possesses, including several lines from Ethiopia that possess a majority OOA2 ancestry (fig. 1*A*). We note, however, that West Africa and Ethiopia also exhibit significant introgression (supplementary table S3, Supplementary Material online), and so shared signals of introgression with each of these Central African ancestries and OOA ancestries are likely not independent. East Africa does not show significant gene flow with any OOA lineage using Patterson's *D*, although we note several lines collected in East Africa are either sister to all of OOA or possess a major OOA ancestry, suggestive of more contemporary patterns of gene flow (fig. 1). Lastly, only West Africa shows evidence of gene flow with OOA1, the ancestry which is most common in North America and the Caribbean (supplementary table S3 and fig. S8, Supplementary Material online; fig. 3*C*). Therefore, we find evidence of more recent as well as likely historical gene flow between several African ancestries and OOA lineages. Although multiple shared signals of introgression likely indicate non-independent incidences of introgression (either via post-introgression migration or introgression to a common ancestor), a lack of shared introgression in other sets of comparisons indicates that rates of gene flow are likely unequal among ancestries (e.g., only West Africa exhibits a significant signal of introgression with OOA1).

It's long been recognized that African ancestry is present in the Caribbean and USA (Yukilevich and True 2008; Duchen et al. 2013; Pool 2015), including a latitudinal cline in African ancestry from the Caribbean through the southeastern USA (Kao et al. 2015; Bergland et al. 2016). However, given the diversity of African ancestries that we describe herein, it is unclear which of these African ancestries has contributed to genetic diversity within the New World. To better understand the source and extent of introgression in the Caribbean and southeastern USA, we integrated genome-wide *D* statistics with windowed analyses using *f*_dM_, which quantifies admixture rates and is better suited to a window approach than Patterson's *D* (Malinsky et al. 2015, 2021). While West Africa exhibited significant introgression with all three OOA ancestries (supplementary table S3, Supplementary Material online), we find that *f*_dM_ is significantly higher with OOA1 than OOA2 (fig. 3*C*). These results indicate that while West Africa likely experienced some amount of gene flow with the common ancestor to all OOA lineages (or each lineage individually), elevated introgression levels between OOA1 and West Africa suggests that OOA lineages have experienced at least partially independent introgression events with West Africa or differential loss of introgressed alleles between OOA ancestries.

We next sought to explore whether West African ancestry can explain the latitudinal cline in African ancestry across the Caribbean and southeastern USA. Using both *K* = 3 and *K* = 14 ancestries, we find significant latitudinal clines in West African ancestry, but no significant clines in other African ancestries (fig. 3*A*, supplementary fig. S9, Supplementary Material online). These results are qualitatively supported by comparable analyses using *f*_dM_ from trios involving ([Ethiopia, West], X), where X denotes a collection site from the Caribbean or the USA. We note that when all samples are included this trend is not significant (*t* = 0.43, *r^2^* = 0.13, *P* = 0.67; Fig 3*B*), as trios where samples from Georgia, North Carolina, or Ithaca were used as P3 exhibit elevated *f*_dM_ with West Africa. When samples from Georgia, North Carolina, and Ithaca were omitted, we recover a marginally significant negative cline in *f*_dM_ across the Caribbean and southeastern USA that mirrors the cline observed in West African ancestry (*t* = −2.32, *r^2^* = −0.66, *P* = 0.05). Together, our results indicate that the Caribbean and USA have either experienced independent gene flow from West Africa relative to other OOA ancestries or there has been less loss of introgressed West African alleles in flies from the Caribbean and southeastern USA than in flies from other OOA ancestries. Clines in West African ancestry may then have been formed via neutral diffusion with northward migration, selection maintaining clines in ancestry, or some combination (see [Bergland et al. 2016] for discussion). Further demographic modeling will be needed to parse the evolutionary forces responsible for these latitudinal clines, as well as more precisely estimate the number and timing of these admixture pulses.

Given the consistent evidence for introgression between West Africa and the OOA1 lineage, we next examined how patterns of introgression vary across the genome. Using the *f*_dM_ analyses from above, we find that chromosomes significantly differ in the extent of introgression (*F* = 163, *df* = 4, *P* < 0.001). We find that the X chromosome exhibited significantly elevated *f*_dM_ relative to the autosomes, while differences among the autosomes were much less apparent (supplementary table S4, Supplementary Material online). However, we note that definitive evidence of increased introgression on the X chromosome is much less apparent when using weighted topologies, as evidenced by a dearth of windows supporting topologies other than the consensus on the X chromosome (fig. 3*D*, supplementary fig. S4, Supplementary Material online). In fact, several previous studies have found lower levels of introgression on the X (Kao et al. 2015; Pool 2015; Bergland et al. 2016). Differences in effective population sizes between the X and the autosomes could be driving some of these discrepancies (e.g., see [Cooper et al. 2015]), alternatively, asymmetric rates of evolution between X chromosomes of the *P1* and *P2* lineage might also contribute to higher rates of erroneously inferred introgression (Xiong et al. 2022). For the autosomes, both *f*_dM_ and analysis of weighted topologies across the genome reveal that peaks of potential introgression appear to be localized and not occurring in extended blocks (fig. 3*D*, supplementary fig. S19, Supplementary Material online). This is perhaps indicative of older introgression with time for haplotypes to be broken apart.

Lastly, we sought to assess how gene flow has uniquely shaped genetic variation in the New World by determining the introgression outliers that are unique to the Caribbean and southeastern USA. We did this by comparing the top 1% of *f*_dM_ windows between West African and both OOA1 and OOA2 ancestries, then subsetting these windows to take only windows that were unique to the West African-OOA1 comparison (see supplementary table S5, Supplementary Material online for full list). We find several genes that may prove fruitful for further examination, including several involved in mating and courtship behaviors, neurological development, and sensory system development and behavior, although none of these gene ontology (GO) categories were significantly overrepresented after appropriate significance testing (see methods for details; supplementary table S6, Supplementary Material online). Notably, flies from West Africa and the Caribbean exhibit unique male courtship and female preference behaviors relative to other OOA and African populations (Yukilevich and True 2008). Thus these introgression outliers may be useful for further genetic dissection of mating behaviors, although much functional work is still needed.

### The Status of Nine Common Inversions

We next used a new linear discriminant method to determine inversion karyotype in our newly sequenced samples (see methods for details, supplementary fig. S14, Supplementary Material online). The frequencies and geographic distributions of the inverted karyotype ranges widely among these nine inversion regions (supplementary fig. S15, Supplementary Material online). For example, *In(2L)t, In(2R)NS, In(3L)OK, In(3R)K*, and *In(3R)P* are all fairly common throughout Africa, particularly in Southern and West Africa, consistent with African origins (supplementary fig. S15, Supplementary Material online; as suggested by Corbett-Detig and Hartl (2012). *In(3L)OK* was most common in samples from Southern Africa, and our results are consistent with findings that this inversion is both common in and unique to Southern Africa, and more common in rural locales than urban locales (supplementary fig. S15, Supplementary Material online). This is evidenced by the commonality of the inverted karyotype in the South1 ancestry group, which consists of more rural samples (supplementary fig. S15, Supplementary Material online; see [Sprengelmeyer et al. 2020]). Phylogenetic trees along inversion regions are largely congruent to trees outside the inversions (supplementary fig. S13 and S16, Supplementary Material online), and consensus trees of inversion regions do not show clustering purely by inversion status, instead clustering more closely by sampling locale. PCAs based on only collinear regions versus based on whole-genome information are also largely similar (supplementary fig. S17, Supplementary Material online). For some inversion regions, this maybe in part driven by their relative rarity (i.e., for *In(3R)Mo, In(1)Be*, and *In(1)A*), for others this may be because the relative frequencies of inversions differ between genetic lineages and many are found in heterozygous form (supplementary fig. S15 and S16, Supplementary Material online). Lastly, these patterns may occur due to rare double crossover events. For example, for the common In*(2L)t* inversion, individuals that are homozygous for the inverted haplotype appear paraphyletic, with one large cluster representing inverted lines from West and East Africa and one representing inverted lines from Southern Africa. Assuming each inversion only evolved once, in the absence of gene flow we would predict individuals that were homozygous for the inversion to be monophyletic, and not cluster by geography. Lastly, we find that inversion regions tend to have higher *π, D*_XY_, and Tajima's *D*, and lower *F*_ST_ than the collinear regions of the genome (supplementary table S8 and fig. S18, Supplementary Material online). Given that each ancestry contained a mixture of karyotypes across inversion regions, these results are expected (supplementary table S8 and fig. S18, Supplementary Material online). Overall, we find several inversions are quite common in samples from remote regions in Southern Africa, suggesting that these inversions may have evolved there. We also find that, although these inversions can distort several population genetic statistics, they do not massively alter patterns of population structure or phylogenetic relationships.

### Distribution of Potential Incompatibilities Throughout *Drosophila Melanogaster*

**Fig. 4.**
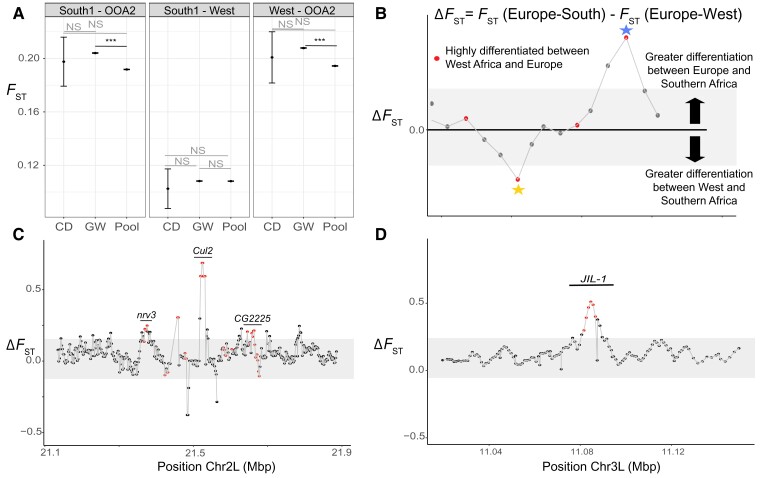
Global differentiation of potential incompatibility loci. (*A*) Distribution of *F*_ST_ for incompatibility loci from Corbett-Detig et al. (2013), Pool (2015), and genome-wide for three comparisons: South1 versus OOA2, West Africa versus South1, and West Africa versus OOA2. (*B*) Cartoon depiction of a scan for incompatibility loci that segregate between African lineages (star below the line) or likely arose during or after the Out of Africa expansion (star above the line). The *Y*-axis denotes the difference in *F*_ST_ between OOA2 and South1 and West Africa and South1, with more positive values indicating that *F*_ST_ is greater between OOA2 and South1 than within Africa. Loci that are highly differentiated between OOA2 and both African lineages are incompatibilities that may have originated during or after an Out of Africa expansion, while loci that are highly differentiated within Africa, but shared between Southern Africa and Europe may represent incompatibilities that originated in Africa. (*C*, *D*) Two zoomed in windows representing one incompatibility pair, as identified by Pool (2015). In panel (*D*) differentiation is elevated between OOA2 and South1 and West Africa and OOA2 (but not West Africa and South1) for loci within the gene *JIL-1*, indicating this allele may be more recently derived in OOA populations. This gene was also originally hypothesized to interact with at least one unidentified gene on chromosome arm 2*L*. In (*C*) we show the corresponding window on 2*L*: again, differentiation is elevated between OOA2 and each of West Africa and South1 (but not within Africa) in several windows within this region containing four genes. Two of these genes are named: *nrv3* and *Cul2*. This approach may provide candidate genes for future functional work.

To understand if and how alleles with negative epistatic fitness effects are structured throughout the range of *D. melanogaster*, we quantified the distribution of candidate incompatibility alleles identified by Corbett-Detig et al. (2013) and Pool (2015). Although we refer to these loci as candidate incompatibilities, they include any loci that are found in repulsion of one another, and may include traditional incompatibilities (i.e., involved in intrinsic postzygotic isolation), loci involved in ecological hybrid breakdown, and loci involved in assortative mating (Schumer and Brandvain 2016). Given their negative fitness effects, we hypothesize that these loci should be geographically structured throughout the range, showing on average higher values of differentiation than the genome-wide average. However, in most comparisons candidate incompatibility loci are no more differentiated than the rest of the genome. When *K* = 3 we find neither set of candidate incompatibility loci were more differentiated than the genome-wide average, and in fact the loci identified by Pool (2015) were less differentiated than the genome-wide average between OOA and each of Central Africa and Southern Africa (supplementary fig. S11, Supplementary Material online). However, *F*_ST_ between Central and Southern Africa was elevated at the loci identified by Pool (2015) compared to the rest of the genome (supplementary fig. S11, Supplementary Material online). For the nine major ancestries, we find only one pair of ancestries with higher *F*_ST_ in candidate incompatibility loci than the rest of the genome (between Ethiopia and HD) for the loci identified by Corbett-Detig et al. (2013) (fig. 4*A*; supplementary fig. S12, Supplementary Material online). For the loci reported by Pool (2015), we find that the majority of comparisons yielded significantly lower *F*_ST_ for candidate incompatibility loci than the genome-wide average (28/36 comparisons; fig. 4*A*; supplementary fig. S12, Supplementary Material online). There are two notable exceptions to this trend. The first is that South2 and each of Ethiopia, and West and East Africa exhibited elevated *F*_ST_ for candidate incompatibility loci relative to the genome-wide average. The second is that two OOA ancestries (OOA1 and OOA2) exhibit elevated *F*_ST_ at candidate incompatibility loci identified by Pool (2015). Thus, while candidate incompatibility loci are more differentiated for a small number of comparisons—predominantly between Southern and Central Africa—they are not broadly differentiated on a global scale among distinct ancestries.

We next assessed whether specific pairs of candidate incompatibility loci showed elevated differentiation, and used patterns of differentiation to characterize their potential geographic origins. Our results are qualitatively similar when using *K* = 3 or the nine major ancestries, and thus we only present the latter below (see supplementary table S7, Supplementary Material online for details). Out of 445 potential incompatibility loci identified by Pool (2015) and 45 potential incompatibility loci identified by Corbett-Detig et al. (2013), we identified only two pairs of interacting candidate incompatibility alleles with high differentiation between West Africa and South1, and low differentiation between South1 and OOA2 (the ancestry most common in Europe); indicative of candidate incompatibilities that may have evolved within Africa. Within these two regions, 25 unique genes are included in the *F*_ST_ outlier windows (supplementary table S7, Supplementary Material online). In contrast, we identified seven pairs of loci with a signature of OOA origin (i.e., low differentiation within Africa, high differentiation between Europe and both West and Southern Africa). We find that the *F*_ST_ peaks within these seven pairs of loci contain 30 unique genes (supplementary table S7, Supplementary Material online). We also note that this approach may prove useful to narrow down candidate loci within the windows originally discovered by Pool (2015) for further functional work (exemplified in fig. 4). Overall, we show that while potential incompatibility loci are not, on average, strongly structured across the globe, some pairs of loci do show high levels of differentiation. These pairs of loci are more highly differentiated between Europe and both Southern and West Africa, but not highly differentiated within Africa; potentially indicative of an origin that occurred during or after an Out of Africa expansion.

## Discussion

The evolutionary history of genetic model systems has been the target of extensive research, including *D. melanogaster* (Pool et al. 2012; Lack et al. 2015; Mansourian et al. 2018; Sprengelmeyer et al. 2020). Nonetheless, sampling gaps across critical regions of the *D. melanogaster* range have left crucial aspects of its history unexplored. By combining existing samples with 223 new samples, 190 of which originate from previously undersampled regions within the ancestral range, we have begun to fill this knowledge gap and report that: individuals from Southern Africa harbor previously undescribed genetic diversity and population structure; *D. melanogaster* has experienced a complex history of gene flow, including both contemporary and historical patterns of admixture within and between continents; several chromosomal inversions differ in frequency between African and non-African ancestries; and potential incompatibility alleles, on average, do not exhibit significant genetic structuring across ancestries. We discuss how this work contributes to our understanding of the complex structuring of genetic diversity within this important model system.

### Population Genetic Structure in the Ancestral Range of *Drosophila melanogaster*

We find several lines of evidence to support the hypothesis that Mopane and Miombo forests in Southern Africa are the likely ancestral range of *D. melanogaster* (as proposed by David and Capy (1988), Lachaise et al. (1988), Pool et al. (2012), Lack et al. (2015), Mansourian et al. (2018), Sprengelmeyer et al. (2020)). First, flies from Southern Africa do not form a monophyletic clade. Instead, our three ancestries from Southern Africa form a nested structure, with some clades being more closely related to all other ancestries of *D. melanogaster* than to more geographically proximal ancestries in Southern Africa. One ancestry, South1, is the most distantly related to all *D. melanogaster*, and largely comprises individuals from rural sampling locales in Mopane and Miombo forests (in agreement with Sprengelmeyer et al. (2020)). These samples may represent extant, pre-commensal populations (as suggested by Mansourian et al. (2018), Sprengelmeyer et al. (2020)). Second, Southern African ancestries generally had the highest diversity, and particularly at *K* = 14, the diversity within South1 was higher than diversity between almost any pair of ancestries, indicating that most diversity with *D. melanogaster* is a subset of diversity within this South1 ancestry. This is in contrast to findings from Sprengelmeyer et al. (2020), who found that flies from Kafue National Park (which is also within the putative ancestral range) exhibited lower diversity than nearby town populations (Sprengelmeyer et al. 2020). These differences may reflect differences in sampling strategies (one sampling locale in Sprengelmeyer et al. (2020) versus seven rural locales herein), but more generally may stem from complex metapopulation dynamics across the ancestral range of *D. melanogaster* (as discussed in Sprengelmeyer et al. (2020)). Under a metapopulation framework, diversity at any given collection locale may be diminished through stochastic events while diversity in the ancestry as a whole is maintained among populations.

We find a strong signal of genetic structure among individuals from the putative ancestral range. In particular, we find two ancestries that were widespread, one of which is more common in flies collected from rural locales and one of which is more common in flies collected from urban locales. One surprising result is the finding of a third major ancestry in Southern Africa that exhibited a distinct evolutionary history relative to either South1 or South2. This third ancestry—which we refer to as HD—is monophyletic on both the autosomes and X chromosome, and comprises a distinct cluster in a whole-genome PCA along PC4. Unlike the other Southern ancestries, HD has substantially reduced nucleotide diversity and elevated Tajima's *D*, indicative of a population bottleneck or recent introgression (or less likely, balancing selection; [Tajima 1989]). Given that seven of nine individuals in this lineage are derived from Harare, one might hypothesize that HD is a product of human-assisted migration into urban centers in Southern Africa. This would be in agreement with previous findings based on microsatellites (Kauer et al. 2003) and whole-genome information from fewer individuals or specific populations (Lack et al. 2015; Medina et al. 2018; Svedberg et al. 2021). However, we do not find genomic evidence of introgression between HD and any OOA ancestry, nor is there reduced divergence between HD and any OOA ancestry relative to the other African ancestries, as would be expected under a scenario of introgression. Assessing the origins and demographic history of the HD lineage—which far more resembles an OOA lineage than any ancestry within Africa—is important to understand the complex demographic history of *D. melanogaster* and better understand factors that have shaped contemporary patterns of diversity and population structure. Although much remains unknown about the ancestry types that we describe—particularly HD—the finding of cryptic structure in one of the most well-studied organisms highlights the need to thoroughly sample ancestral ranges when quantifying diversity and structure.

While our Patteron's *D* analyses do not strongly support a scenario of historical gene flow between most Southern African ancestries and other ancestries, mismatches between major ancestries and geographic sampling locations may provide insight into contemporary gene flow. When the major ancestry does not correspond with geographic location we infer that these individuals represent recent migration events which can potentially provide insight into the directionality of contemporary gene flow. These mismatches were quite common- particularly between flies collected in South Africa and both OOA and Ethiopia. In line with this observation, recent ancestry-based analyses have found evidence of a single, recent pulse of admixture from cosmopolitan flies into several African *D. melanogaster* populations, including in Ethiopia and South Africa (Lack et al. 2015; Medina et al. 2018), including an example of adaptive introgression of genes conferring insecticide resistance in South Africa (Svedberg et al. 2021). Better understanding the timing and extent of migration between Eurasia and both Central and Southern Africa will help to clarify the connectivity and population genetic structure exemplified herein. Moreover, assessing whether patterns of admixture and migration are more readily apparent in flies from more urban locales can better address the role of humans in *D. melanogaster* migration as well as highlight other potential examples of adaptive introgression in a human commensal environment.

### Patterns of Transcontinental Gene Flow

It has long been recognized that flies from the Caribbean and southeastern USA possess both European and African ancestry (Pool et al. 2012; Duchen et al. 2013; Kao et al. 2015; Lack et al. 2015; Pool 2015; Bergland et al. 2016). Here we provide further resolution to the dynamics of this admixture zone by demonstrating that Central African—and in particular West African—ancestry is the most likely contributor of African ancestry in this contact zone. This is exemplified using Patterson's *D* and *f* statistics, by being the only African ancestry to exhibit a significant signal of introgression with the ancestry that is most common in the Caribbean and southeastern USA (OOA1). Further, West African ancestry is the only ancestry type to show a clinal gradient of prevalence across the Caribbean and North America, which is an important pattern structuring admixture in these populations.

Not only is there a cline in African ancestry but there is also a behavioral cline in populations from the eastern shore board of the USA and the Caribbean. Caribbean females will mate with West African males but show levels of premating isolation and mate discrimination with flies from Europe and Zimbabwe (Yukilevich and True 2008). Previous work has also shown that flies from West Africa exhibit unique mating behaviors that confer reproductive isolation with flies from both Europe and Zimbabwe (Capy et al. 2000; Haerty et al. 2002; Yukilevich and True 2008). Combined these observations support our genetic analysis that gene flow between flies from West Africa and the Caribbean may underlie this shared behavior, and we were able to leverage this information to identify loci that may contribute to these behaviors. We find five genes in the top 1% of introgressing loci between West Africa and OOA1 are related to mating and courtship behaviors: *egh*, *dlg1*, *btv*, *CaM*, and *lov.* Expression of *egh* and *dlg1* both regulate the amount of time spent courting, with expression of *egh* in particular being required for male courtship to occur at all (Mendoza-Topaz et al. 2008; Ellis and Carney 2011). Males with nonfunctional copies of *lov* exhibit passive courtship, often failing to perform a full sequence of courtship behaviors and/or not directing their attempts towards females (Bjorum et al. 2013)*. btv* affects courtship song, wherein mutants are largely deaf and have malformed chordotonal organs which prevents them from singing (Eberl et al. 1997; Tauber and Eberl 2001). *CaM* influences olfactory responses by trafficking odorant receptors; a key signal facilitating courtship interactions (Bahk and Jones 2016). While functional validation will be required to test the role of each of these genes in the shared male mating behaviors between West Africa and the Caribbean, this work provides a useful step forward in understanding how patterns of gene flow have shaped phenotypic variation across the range of *D. melanogaster*.

We also find several genes involved in other biological processes, including immune function, taxis and locomotion, and neurological development. Whether any of these introgressed genes contribute to the well-described clinal and season variation across the eastern USA (Bergland et al. 2016; Machado et al. 2016; Machado et al. 2021) remains unknown. However, we note that ∼10% of genes found in the top 1% of *f*_dM_ outliers are highly differentiated across the eastern USA (i.e., 21/255 genes in our *f*_dM_ outlier analysis were found as *F*_ST_ outliers in Bergland et al. (2016). This work highlights a potential role of introgressed alleles as a source of genetic variation which can then be shaped by spatially or temporally varying natural selection. Overall, this work helps to disentangle the complex patterns of gene flow throughout the range in *D. melanogaster* and identify how modern genetic diversity is shaped by historical patterns of migration.

### Candidate Incompatibility Loci are not Strongly Structured Throughout the Range

Previously identified candidate incompatibility loci are known to segregate in *D. melanogaster* populations and have negative fitness effects (Corbett-Detig et al. 2013; Pool 2015). While we *a priori* hypothesized that candidate incompatibility loci should be highly structured throughout the range of *D. melanogaster*, we find no evidence to support this claim. Instead, candidate incompatibility loci are largely not differentiated between ancestries. While loci are, on average, not strongly structured throughout the range of *D. melanogaster*, we do find nine putatively interacting pairs of loci that exhibit elevated differentiation. Of these nine pairs, seven pairs show high differentiation between OOA and both West and Southern Africa. These patterns are consistent with a scenario in which at least one interacting allele involved in potential incompatibilities evolved after the out of Africa expansion. In contrast, the remaining two pairs of loci show strong signals of differentiation between West and Southern Africa, in line with the hypothesis that these candidate incompatibilities may have evolved before the out of Africa expansion. While much remains unknown about the majority of candidate incompatibility alleles first identified by Corbett-Detig et al. (2013), Pool (2015), the result that these loci are not highly differentiated throughout the range of *D. melanogaster* has significant implications for epistatic fitness variation within natural populations.

## Conclusions

Despite being one of the best studied organisms, the full range of genetic diversity of *D. melanogaster* is still being revealed. In this study, we complement existing samples with 223 new lines, 190 of which originate from the ancestral range of *D*. *melanogaster.* We demonstrate that this range harbors significant genetic diversity and structure, with different ancestries exhibiting different evolutionary histories. While our results build upon a growing exploration of the natural history and natural genetic diversity within this key model system, our findings also raise new questions. In particular, assessing the role of natural selection in the patterns we demonstrate herein will present a compelling next step.

## Materials and Methods

### Sampling Sequencing and Variant Calling

We created 244 new *D. melanogaster* isolines from 339 wild collected females derived from seven novel locations in Zambia, Namibia, and Zimbabwe using a similar approach to ([Sprengelmeyer et al. 2020]; see supplementary table S1, Supplementary Material online for sampling locations; see supplementary methods, Supplementary Material online for details). We then sequenced whole genomes for 190 of these accessions plus 33 advanced generation inbred lines at the University of North Carolina (UNC) School of Medicine (see supplementary table S1, Supplementary Material online and supplementary methods, Supplementary Material online for details). Our re-sequenced lines were paired with whole genome sequences for an additional 589 isolines via NCBI SRA, including 266 lines from outside of Africa and 323 from within Africa (Pool et al. 2012; Lack et al. 2015) (see supplementary table S1, Supplementary Material online for details). Although we do not include all previously sequenced lines, our subsample is a representative subsample and includes accessions from all previously sequenced populations reported in Pool et al. (2012), Lack et al. (2015, 2016).

We generated a vcf of all 803 *D. melanogaster* plus 13 *D. simulans* genomes using a standard pipeline that followed best practices (see supplemental methods and supplementary table S1, Supplementary Material online for details). The resulting VCF was filtered so that indels were removed, and only invariant and biallelic sites with a minimum quality score of 30, minimum coverage of 5X, minimum genotype quality of 30, a maximum of 25% missing data were kept.

### Assessing Karyotypes in New Samples

Large chromosomal inversions are known to segregate within *D. melanogaster*, and these inversions can have extended effects on patterns of differentiation across chromosome arms (Corbett-Detig and Hartl 2012). We therefore sought to call karyotypes across our newly generated fly lines for nine large and relatively common inversions. To do so, we used an LDA approach based on the ancestry proportions generated by *PCAngsd* (Meisner and Albrechtsen 2018). Briefly, for each chromosomal inversion, we used *Angsd* to generate a beagle file that contained genotype likelihoods for only sites between the proximal and distal breakpoints for each inversion. We retained sites that had a minimum mapping quality (minMapQ) of 30, a minimum quality (minQ) of 20, and a genotype information for at least 90% *D. melanogaster* individuals. Then, using *PCAngsd*, we inferred *K* (the number of distinct ancestry types) for each inversion region and performed an LDA analysis on the ancestry proportions. As inversions suppress recombination in heterozygotes, they can create long blocks of linkage disequilibrium in natural populations (as found in Corbett-Detig and Hartl (2012), Twyford and Friedman (2015)), and thus the expectation is that major PCs or ancestry types should largely describe these extended haplotypes (demonstrated in Berg et al. (2017), Battey et al. (2021), Funk et al. (2021)). In the case of *D. melanogaster*, inversion karyotypes have been previously described for the vast majority of the sample included herein (Corbett-Detig and Hartl 2012; Lack et al. 2015). To leverage this information, we trained and tested each model using a restricted dataset of only individuals with known karyotype. Each model used 75% of individuals with known karyotype to train the model and 25% of individuals with known karyotype to test each model. Each model performed quite well, with an average error rate of 3.5% (range: 0–8%). We then applied the model to the full dataset to predict karyotypes for all newly sequenced lines. Although homozygous genotypes were relatively easy to detect visually (supplementary fig. S14, Supplementary Material online), we avoid definitively calling heterozygous genotypes, unless the LDA showed clear separation of all three inversion genotypes (i.e., *In*(2R)NS*, In*(3L)OK*, In*(3L)P; supplementary fig. S14, Supplementary Material online). Nonetheless, we performed all analyses using either all individuals in only collinear regions (>100 KB away from inversion breakpoints) or only individuals with high confidence standard karyotypes in inverted regions unless otherwise noted. This approach allowed us to minimize the impact of inversion polymorphism on patterns of population structure and gene flow.

### Lineage Relationships Population Structure PCA and Phylogenetic Reconstruction

To better understand the relationships among a global sampling of *D. melanogaster*, we constructed maximum likelihood (ML) phylogenies in windows across the genome using *iqtree* version 1.6.12 (Nguyen et al. 2015; Kalyaanamoorthy et al. 2017; Hoang et al. 2018). We generated ML trees for non-overlapping 100 KB windows using the model-finder and ultra-fast bootstrap approach with 1,000 bootstraps. We then used the resulting ML trees from regions of the genome excluding the nine common inversions (as well as 100 KB from both distal and proximal breakpoints) in *D. melanogaster* as input for *ASTRAL v5.1.1* (Zhang et al. 2018) in generating a consensus tree for the autosomes and X chromosome independently (supplementary fig. S2, Supplementary Material online). Finally, we generated consensus trees for each of the nine inversion regions by running *ASTRAL* on all trees within inversion breakpoints.

To characterize fine-scale population genetic structure, we performed *K*-means clustering analysis and PCA using *PCAngsd* (Meisner and Albrechtsen 2018) and *NGSAdmix* (Skotte et al. 2013) for the *D. melanogaster* samples. *PCAngsd* uses genotype likelihoods to first perform a genome-wide PCA, then assess population structure with the number of ancestry types (*K*) defined as the number of significant PCs + 1. In contrast, *NGSAdmix* functions similarly to a typical *K-means* clustering program, wherein we computed the likelihood for a range of values of *K.* In our case, we estimated the likelihood of five replicate runs of *K* for each value from 2 to 15. We then used *CLUMPAK* to estimate the best *K* (Kopelman et al. 2015). For both approaches, we generated a.*Beagle* file using *Angsd* (Korneliussen et al. 2014) that included only collinear regions in the genome (i.e., 100 KB away from inversion breakpoints), and sites with a minimum mapping quality (minMapQ) of 30, a minimum quality (minQ) of 20, and a genotype information for at least 90% *D. melanogaster* individuals were retained. This resulted in the inclusion of 332,296 sites across 803 individuals.

*NGSAdmix* and *PCAngsd* differed in the inferred number of unique ancestries, with *NGSAdmix* inferring *K* = 3 and *PCAngsd* inferring *K* = 14. Although they differed in the total number of ancestries, we note that, in general, *PCAngsd* merely divided the *NGSAdmix* inferred ancestries to reveal further fine-scale structure (see fig. 1 for details). For the *K* = 14 ancestries, we identified nine ancestries that are common, and are commonly the major ancestry within an individual (i.e., the ancestry that is >50% within an individual). Additionally, when individuals are defined based on these major ancestries, most major ancestry types are largely monophyletic. Given these observations, we defined nine major ancestries from the *K* = 14 analyses. Three ancestries are most common in Southern Africa: one is largely found in individuals from Harare, Zimbabwe (we refer to this as HD), the other two include individuals from many collection locales (we refer to these as South1 and South2), but we note that South1 tends to include more rural samples and is sister to all other lines studied herein. Three major ancestries largely include lines from Out of Africa (OOA). One of these is mainly restricted to Beijing, one largely contains individuals from Europe, Egypt, and Tasmania (OOA2), and one largely contains individuals from the southeastern USA and the Caribbean (OOA1). The final three ancestries are mainly found in Central Africa, with a unique ancestry being most common in Ethiopia, all of West Africa, and East Africa (minus Ethiopia). All subsequent analyses were performed on populations defined by ancestry, with each analysis performed for *K* = 3 ancestries and the nine major ancestries groups described above.

To estimate pairwise measures of divergence and differentiation between these ancestries and nucleotide diversity within ancestries, we used *pixy* (Korunes and Samuk 2021) to calculate *F*_ST_, *D*_XY_, and *π* in 1 KB windows across the genome using with default filtering expressions (i.e., DP≥10, GQ≥20, RGQ≥20). For this analysis we used an all-sites VCF to include invariant sites. We also calculated Tajima's *D* using *VCFTools* v.01.15 (Danecek et al. 2011), and estimated the Site-Frequency Spectra (SFS) using *SweeD* v.3.2.4 (Pavlidis et al. 2013) for each ancestry type.

### Introgression Analyses

To estimate broad patterns of gene flow between genetic lineages of *D. melanogaster,* we first calculated Patterson's *D* and *f*_G_ (a Patterson's *D* derivative which more accurately estimates the proportion of the genome experiencing introgression [Martin et al. 2015]) using *Dsuite* (Malinsky et al. 2021) with *D. simulans* as the outgroup for all possible trios, given the following phylogeny: ([{OOA, ((East, West), Ethiopia)}, HD], Southern). Because the OOA lines represent three distinct and non-monophyletic major ancestries, “OOA” in the phylogeny above could represent OOA1, OOA2, or Beijing. Similarly, “Southern” in the phylogeny above could represent either the South1 or South2 major ancestry group. In total, we present results for 65 unique and phylogenetically accurate trios. Significance of Patterson's *D* was determined using a standard block jackknife procedure (Malinsky et al. 2021), we then implemented a Bonferroni correction for multiple testing, which resulted in 17 significant comparisons (supplementary table S3, Supplementary Material online).

#### Focal Trios

In specific cases, we also quantified differences in the extent of introgression between genetic lineages using *f*_dM_ in non-overlapping 20 SNP windows. *f*_dM_ is an *f* statistic derivative that is more appropriate for windowed analysis and provides a more accurate estimate of the proportion of the genome that has experienced introgression than Patterson's *D* (Malinsky et al. 2021). Finally, we bolstered our introgression analyses by assessing heterogeneity in the genome in the relationships between potentially introgressing groups by calculating tree topology weights using *twisst* (Martin and Van Belleghem 2017). For each focal trio (outlined below), we calculated the topology weight at each non-overlapping 100 KB window for trees comprising four groups. While *f*_dM_ calculates the proportion of shared derived variants between non-sister lineages, *twisst* assesses the proportion of topologies that fit particular phylogenetic relationships. These analyses thus provide complimentary, but uniquely informative quantifications of introgression.

The first set of analyses sought to assess differences in allele sharing among West Africa and various Southern African ancestries. For the *f*_dM_ analyses, we calculated *f*_dM_ using the following phylogeny: ([{ET, West}, X], simulans), where X could denote each of the three Southern African ancestries (HD, South1, and South2). To assess differences in the extent of derived allele sharing between West Africa and each Southern African ancestry, we performed an ANOVA with Type III SS using *f*_dM_ as the response variable and P3 (i.e., HD, South1, and South2 as the levels), chromosome, and their interaction as independent variables. We also used *twisst* to assess heterogeneity in phylogenetic relationships across the genome, with ([{ET, West}, HD], South1) being the consensus tree. Because only West and HD exhibited a significant Patterson's *D* and our goal was to assess potential signals of introgression between West Africa and HD, we only performed this analysis with HD as sister to the Central African clade.

For the second set of analyses, we sought to further explore potential signals of introgression between West Africa and OOA ancestries. For these analyses, we used South1 as the outgroup to better polarize SNPs. We calculated *f*_dM_ in 20 SNP windows across the genome for the following phylogeny ([{East, West}, X], South1), where X could denote OOA1 or OOA2. We then asked if the value of *f*_dM_ differed between these two comparisons using an ANOVA with type III SS, with *f*_dM_ as the response variable and P3 (i.e., OOA1 or OOA2 as the levels), chromosome, and their interaction as the independent variables. We also completed these analyses with ET as P1, but as these results of these analyses did not differ, we present only the findings in which East was used as P1. To bolster these results, we then tested the following phylogenetic relationship: ([{OOA2, OOA1}, West], South1) in *twisst,* and asked if there was a surplus of windows in which West Africa and OOA1 were sister, relative to West Africa and OOA2 using an ANOVA with type III SS, with the number of windows supporting each relationship as the response variable and the comparison (i.e., West sister to OOA1, and West sister to OOA2 as the levels), chromosome, and their interaction as independent variables. Under a model of either equivalent introgression or ILS, there should be no difference in either the number of windows which show West Africa and either OOA ancestry as sister or the values of *f*_dM_ with either OOA ancestry as P3. In both cases, we find that West Africa and OOA1 exhibit significantly more allele sharing than West Africa and OOA2. To further assess clines in West African ancestry across the Caribbean and the southeast USA, we calculated *f*_dM_ again, but instead of grouping all collection locales into one population based on major ancestry, we used each collection locale as P3.

Lastly, we sought to assess whether genes involved in particular biological functions were more likely to introgress between West Africa and OOA1. For these analyses, we took the top 1% of *f*_dM_ windows between West Africa and each OOA ancestry, then parsed the windows that were unique to the West Africa-OOA1 comparison. We then performed an overrepresentation test using PANTHER v.17.0 with an FDR cutoff of 0.05 (Thomas et al. 2003; Mi et al. 2020). Because genes can vary in length and GO categories are not randomly distributed across the genome, we created a null distribution for the expected occurrence of different GO categories in an *f*_dM_ outlier analysis by permuting our outliers 10K times (similar to Pool et al. (2012), Sprengelmeyer et al. (2020)). For each permutation, a random 1% of windows analyzed for introgression were selected as outliers, unique genes overlapping these windows were extracted, and the number of genes in each of 7,791 GO categories analyzed were calculated. Finally, *Z*-scores for observed numbers of genes compared to the null distribution were calculated to determine significance for each GO category, with Benjamini–Hochberg corrected *P*-values under 0.05 taken as significant.

### Determining the Global Distribution of Previously Identified Incompatibilities

Lastly, we characterized patterns of differentiation for loci that have previously been implicated in two studies of genetic incompatibilities with *D. melanogaster* (Corbett-Detig et al. 2013; Pool 2015). First, Corbett-Detig et al. (2013) used a global panel of *D. melanogaster* inbred lines to create synthetically admixed populations from a series of round-robin matings followed by continual inbreeding. This design enabled the identification of pairs of alleles that appear less frequently than expected under random mating and Mendelian segregation in their final recombinant inbred line population (i.e., Genotype Ratio Distortion). Using a similar premise, but in a naturally admixed population, Pool (2015) used patterns of linkage disequilibrium in the southeastern USA to assess pairs of alleles that occur together less frequently than expected based on their allele frequencies (i.e., Ancestry Disequilibrium). Pool (2015) also determined that many of these loci were highly differentiated between Africa and Europe, using populations from West Africa and France, respectively.

Elevated differentiation of these candidate incompatibility alleles between West Africa and France may stem from multiple evolutionary scenarios, and differentiating these scenarios can help elucidate the geographic distribution and potential origins of candidate incompatibilities within *D. melanogaster*. Here, we aim to differentiate two potential scenarios: First, potential incompatibilities between Europe and Africa may have arisen with or after the Out of Africa expansion. Under this scenario, we predict that differentiation at potential incompatibility loci should be low between genetic lineages in Africa, but high between Europe and all African populations. Second, it is also plausible that candidate incompatibilities between Europe and West Africa originated in Africa, with shared ancestry or subsequent introgression explaining allele sharing between Europe and Southern Africa. Under this scenario, we expect that differentiation should be high between West Africa and both Europe and Southern Africa, but relatively low between Europe and Southern Africa.

To differentiate these scenarios, we used the *F*_ST_ windows from above for all pairwise comparisons between our nine major ancestries. We also performed these analyses using *K* = 3 ancestries for comparison. These two scales of analyses broadly agree, and so we focus on the nine major ancestries for clarity's sake. We first ask if potential incompatibility loci have elevated divergence relative to the whole genome for any comparison using an ANOVA with Type III SS using the *car* library in *R* (Fox and Weisberg 2018) for each pair of ancestries. Specifically, *F*_ST_ was the dependent variable, and locus-type (genome-wide, loci identified by Corbett-Detig et al. (2013), or loci identified by Pool (2015)), was the independent variable. We then used pairwise *t*-tests to assess significance between the locus-type levels. Second, we assessed the history and distribution of individual pairs of loci that are predicted to interact and differentiate the two evolutionary scenarios outlined above. For these analyses, we identified potential incompatibility pairs in which both interacting loci were either highly differentiated between West Africa and OOA2 as well as West Africa and Southern Africa (indicative of potentially older incompatibility loci), as well as pairs of interacting loci that were highly differentiated between OOA2 and each of West Africa and Southern Africa (potentially indicative of new incompatibility loci). For these analyses, we define highly differentiated loci as those with *F*_ST_ values within the top 2.5% of *F*_ST_ for that population pair.

## Supplementary Material

msac223_Supplementary_Data

## Data Availability

All new raw sequence data are available on the NCBI SRA under the project number PRJNA880311. Current SRA codes are noted in supplementary table S1, Supplementary Material online.
