# Supplementary material for: Patterns of Population Structure and Introgression Among Recently Differentiated Drosophila melanogaster Populations
Source: Mol Biol Evol. 2022 Oct 17;39(11):msac223. doi: 10.1093/molbev/msac223 (PMC9641974; doi:10.1093/molbev/msac223)
Supplement: msac223_Supplementary_Data [file msac223_supplementary_data.zip › Coughlan et al.2022.MBE.supmat.pdf]

SUPPLEMENTAL METHODS

Sampling

We collected *D. melanogaster* from seven locations in Zambia, Namibia, and Zimbabwe using a similar approach to previously described efforts ((Sprengelmeyer et al. 2020); see Table S1 for sampling locations). Our approach differed from other samplings in that we used multiple potential substrates. Each trap consisted of buckets adjacent to each other and separated by about 50cms. The buckets were filled with mashed bananas (purchased locally), Marula (*Sclerocarya birrea*) or muzinzila fruits (*Berchemia discolor).* In all cases, we removed the husk of the fruit, added yeast (Red Star Active Dry Yeast - 16 oz. #201265), and allowed them to ferment for about 24 hours. These traps were put underneath trees. We collected all flies in the bucket using a sweeping net (BioQuip; Rancho Domingo, CA) after 24, 48, and 72 hours. We then netted and aspirated flies with a pooter (1135A Aspirator–BioQuip; Rancho Domingo, CA)) and anesthetized within 20 minutes of collection using FlyNap (triethylamine, Carolina Biological Supply Co.). Females and males were separated. Males and individuals from other species were placed in ethanol; *D. melanogaster* females were placed in 30mL plastic vials with cornmeal food and allowed to oviposit. Of 339 collected females, we were able to establish 244 isofemale lines (i.e., shelf-stable lines derived from a single matriarchal lineage).

DNA extraction and sequencing

We extracted DNA from 20 individuals from each of the 223 unique isofemale lines using a Gentra Puregene Tissue Kit (Qiagen, Valencia, CA, USA) following the recommended tissue protocol with volumes of reagents as suggested for processing 5 - 10 mg of tissue (see Table S1 for collection details). To prepare the genomic DNA libraries we used KAPA HyperPrep kits (Roche Sequencing, Pleasanton, CA) with a target fragment size of 300-500 bp at the University of North Carolina (UNC) School of Medicine's high-throughput sequencing facility. Next, we pooled individually barcoded libraries into groups of ~10 individual isoline libraries and each pool was sequenced on either a single lane of an Illumina HiSeq 4000 or a Noveseq6000S4XP platform, in both cases generating paired-end 150 bp reads. This sequencing strategy yielded between 2.6-23.1 billion bp of raw sequence data for each individual (See Table S1 for coverage information).

Public data

We obtained whole genome sequences for an additional 589 isolines via NCBI SRA. Of these publicly available genomes, 266 lines from outside of Africa and 323 from within Africa, 143 of which are derived from Southern Africa (Pool et al. 2012; Lack et al. 2015) (see Table S1 for details). Although we do not include all previously sequenced lines, our subsample is a representative subsample and includes accessions from all previously sequenced populations reported in (Lack et al. 2015).

Variant calling

We aligned 803 genomes of *D. melanogaster* to the *D. melanogaster* v6.32 reference genome (dos Santos et al. 2015) using *bwa mem* function (Li and Durbin 2009). We

then used *Picard Tools* (http://broadinstitute.github.io/picard/) to clean, sort and dedupe individual files before individually genotyping them in *GATK4.2.4.1* (McKenna et al. 2010) with the *HaplotypeCaller* function. All samples were then jointly genotyped in *GATK4.2.4.1* using the *GenotypeGVCfs* function, following *GATK* best practices (McKenna et al. 2010). The resultant VCF was filtered so that indels were removed, and only biallelic sites with a minimum quality score of 30, minimum coverage of 5X, minimum genotype quality of 30, a maximum of 25% missing data were kept. For some analyses, we additionally removed five individuals with poor quality genomes (e.g. less than 5X average coverage). For analyses requiring an outgroup (such as our phylogenetic reconstruction, outlined below), we also included 13 *D. simulans* (see Table S1 for SRA accession numbers).These sequences were processed as above, and VCF files were merged using *bcftools merge* function (Li et al. 2009).

SUPPLEMENTAL TABLES:

Table S1: Sample information for all individuals in this study. For each accession, the unique ID (sample), species (each are species names of the genus *Drosophila*), NCBI SRA code (new samples will be given a unique code upon data upload), geographic coordinates of collection, and several classifications of genetic lineage based on collection locale, K=3 assignment, and K=14 assignment, the given and inferred karyotypes for nine common chromosomal inversions, and the average depth of coverage are given.

Table S2: F_{st} (blue; lower triangle), D_{xy} (green; upper triangle), and π (diagonal) for all geo-genetic lineages of *D. melanogaster* and *D. simulans (simulans)*. Population codes: Ethiopia= Ethiopia, East= East Africa, West= West Africa, South= Southern Africa, HD= Harare Distinct, OOA= Out of Africa, Beijing=Beijing.

Table S3: *D* statistics of all trios based on K-14 major ancestries. Population codes: Ethiopia= Ethiopia, East= East Africa, West= West Africa, South= Southern Africa, HD= Harare Distinct, OOA= Out of Africa, Beijing=Beijing.. Note that OOA1 is the ancestry type most common in the Caribbean and North America, while OOA2 is more common in Europe, Tasmania, and Northern Africa. Bolded *D* statistics indicate significance after Bonferroni correction.

Table S4: Mean f_{dM} values for each chromosome arm by each focal comparison. Significant differences between chromosome arms for each comparison are indicated by superscript letters.

Table S5: Introgression Outliers. Top 1% of f_{dM} outliers which were unique to OOA1-West. Genes within the outlier window are given.

Table S6: PANTHER analyses on the top 1% fdM outliers which were unique to OOA1-West. For the over/under column, O= over, U=under. Significant After Permutation refers to a separate significance test to account for gene length and

position.

Table S7: Candidate incompatibilities with F_{st} outliers. A list of potential incompatibilities identified by (Pool 2015) and (Corbett-Detig et al. 2013) in which both interacting loci show signatures of older or more recent derivation (denoted in the Popgen signature). Window positions were translated between V.5 and V.632 of the *D. melanogaster* reference genome, as different reference genomes were used between (Pool 2015) and the current study. As some windows identified by Pool contain multiple F_{ST} outliers, the number of outliers contained for each locus window, plus the genes that fall within that outlier are listed. We completed these analyses for both *K*=3 and *K*=14 ancestries, and differences between ancestry comparisons are denoted.

Table S8: Diversity, divergence, and differentiation for all locus types and all ancestries (*K*=14). (A) Average pairwise divergence and differentiation for all possible ancestry combinations (plus *D. simulans*) for the whole genome (WG), just collinear regions (COL) and nine common inversion regions (INV). (B) Average diversity for each of nine major ancestry types and *D. simulans.*

SUPPLEMENTAL FIGURES:

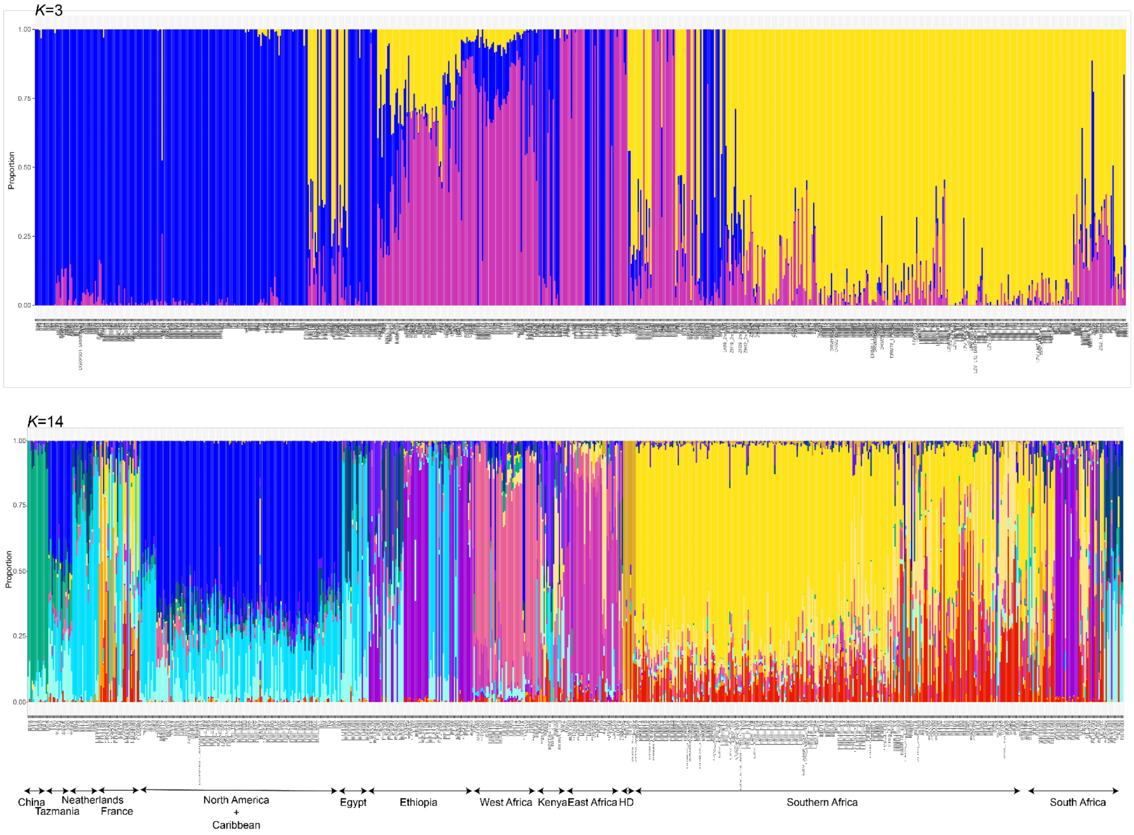

Fig. S1- Ancestry designations for (A) *K*=3 and (B) *K*=14 for all individuals.

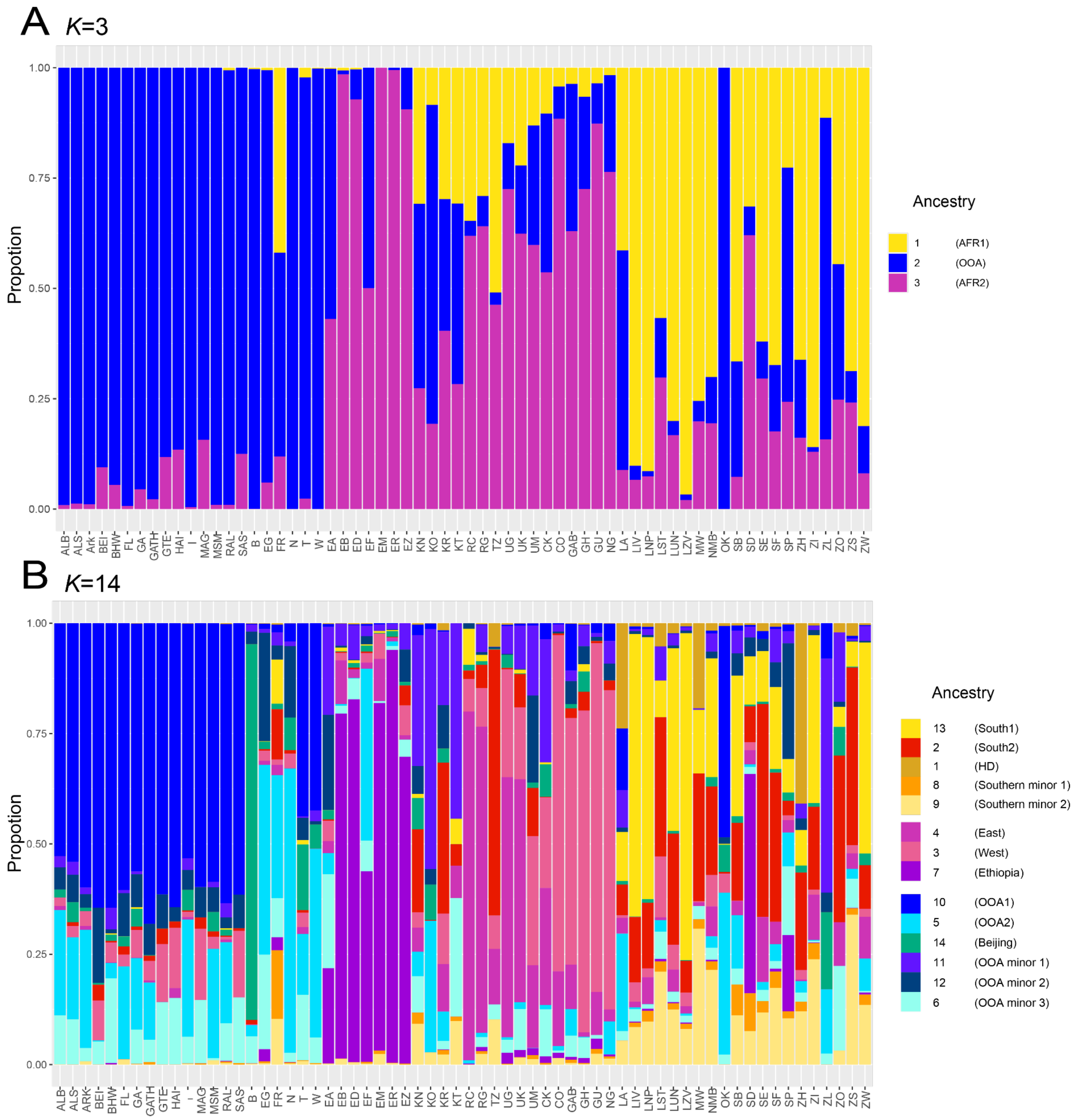

A K=3
Propotion
1.00
0.75
0.50
0.25
0.00
ALB ALS Ark BEI BHW FL GA GATH GTE HAI I MAG MSM RAL SAS B EG FR N T W EA EB ED EF EM ER EZ KN KO KR KT RC RG TZ UG UK UM CK CO GAB GH GU NG LA LIV LNP LST LUN LZV MW NMB OK SB SD SE SF SP ZH ZI ZL ZO ZS ZW
Ancestry
1 (AFR1)
2 (OOA)
3 (AFR2)
B K=14
Propotion
1.00
0.75
0.50
0.25
0.00
ALB ALS ARK BEI BHW FL GA GATH GTE HAI I MAG MSM RAL SAS B EG FR N T W EA EB ED EF EM ER EZ KN KO KR KT RC RG TZ UG UK UM CK CO GAB GH GU NG LA LIV LNP LST LUN LZV MW NMB OK SB SD SE SF SP ZH ZI ZL ZO ZS ZW
Ancestry
13 (South1)
2 (South2)
1 (HD)
8 (Southern minor 1)
9 (Southern minor 2)
4 (East)
3 (West)
7 (Ethiopia)
10 (OOA1)
5 (OOA2)
14 (Beijing)
11 (OOA minor 1)
12 (OOA minor 2)
6 (OOA minor 3)

Fig. S2- Average ancestries for (A) *K*=3, and (B) *K*=14 per sampling locale.

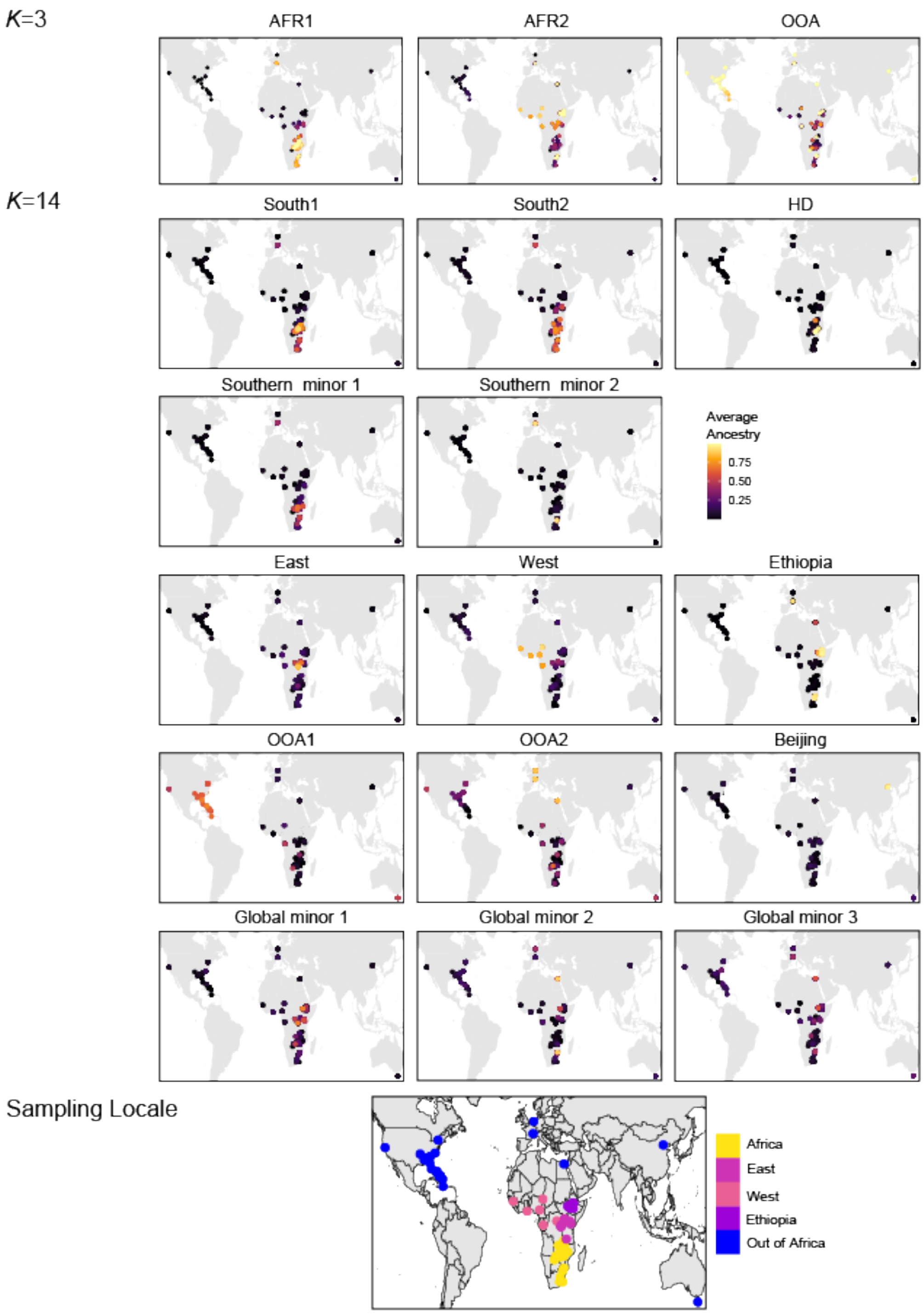

Fig S3 - Distribution of ancestries and sampling designations across the globe. Average

ancestry per population and population location for K=3 clusters (top), K=14 clusters (middle) and geographic designations of sampling location (bottom).

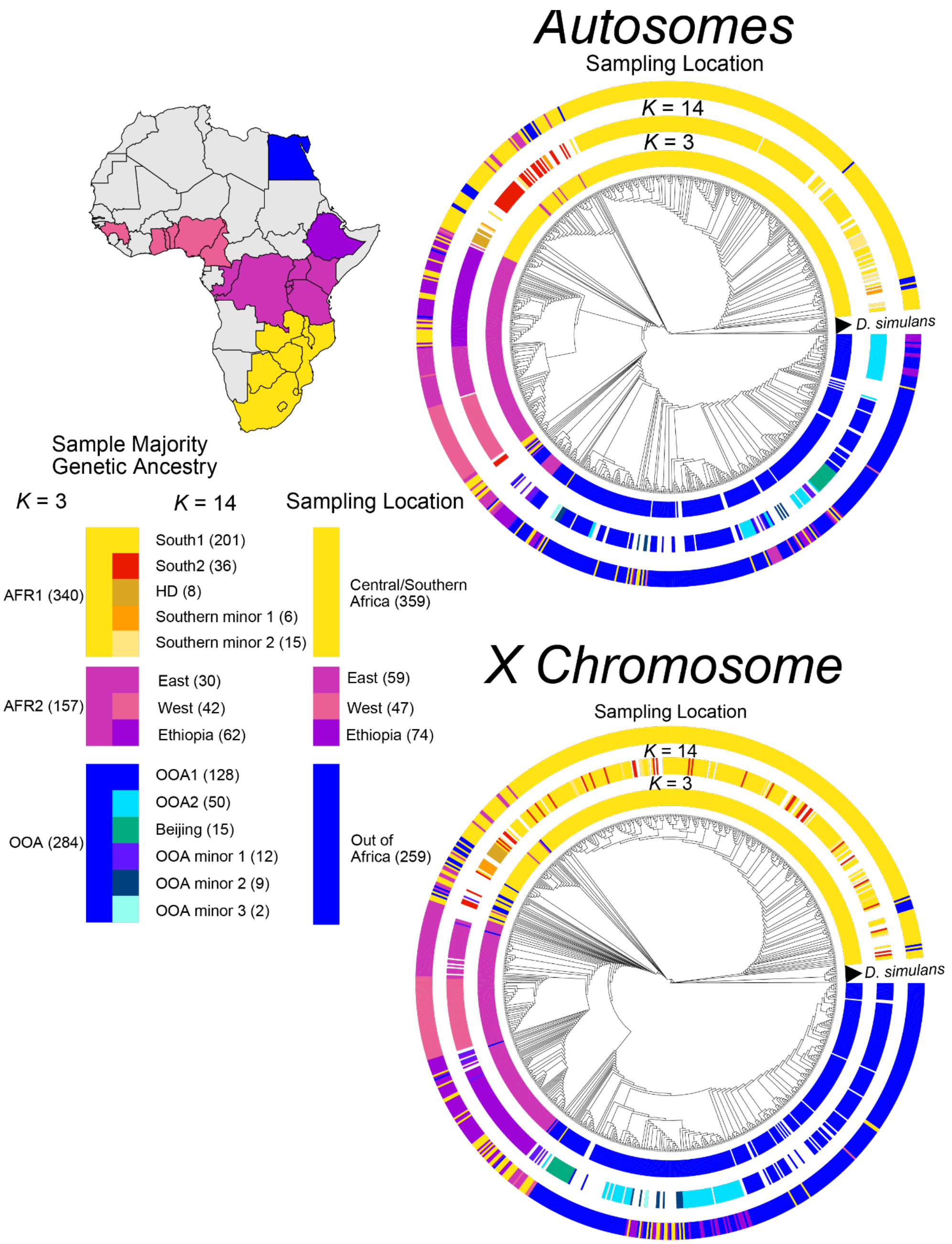

Figure S4: Full ML phylogeny of all samples based on collinear regions of (A) autosomes and (B) the *X* chromosome only. As in Figure 1, the inner ring represents the majority ancestry of each sample when K=3, middle ring for K = 14 and outer ring

represents sampling location, colored in on the map of Africa (with all out of Africa samples also being blue).

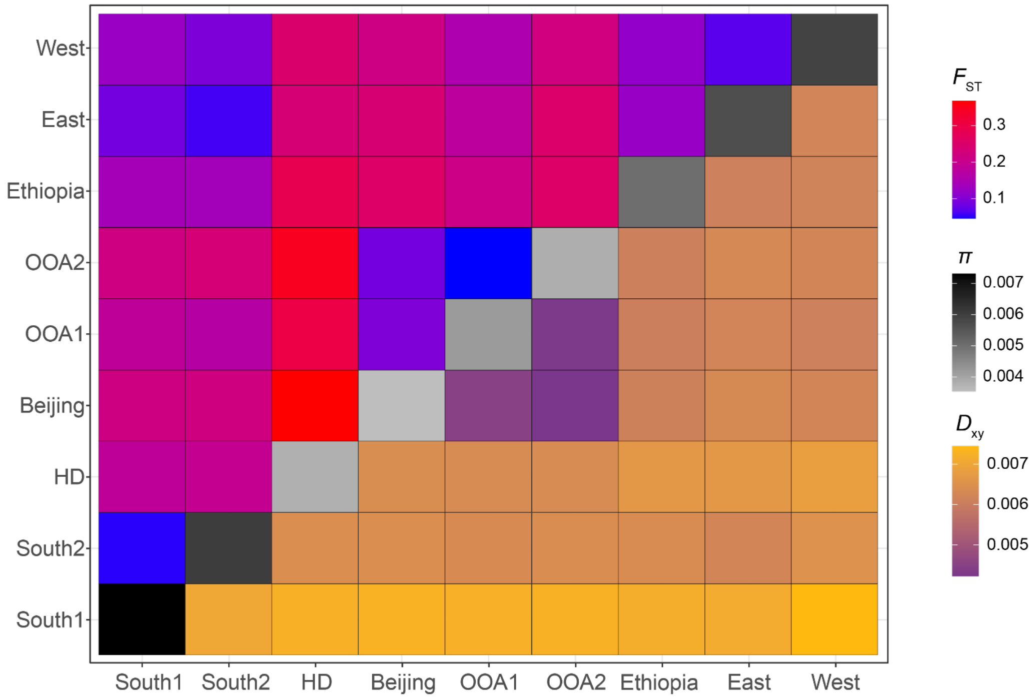

Fig. S5- All pairwise measures of diversity, differentiation, and divergence for *K*=14 ancestries are shown on the diagonal, upper triangle and lower triangle respectively.

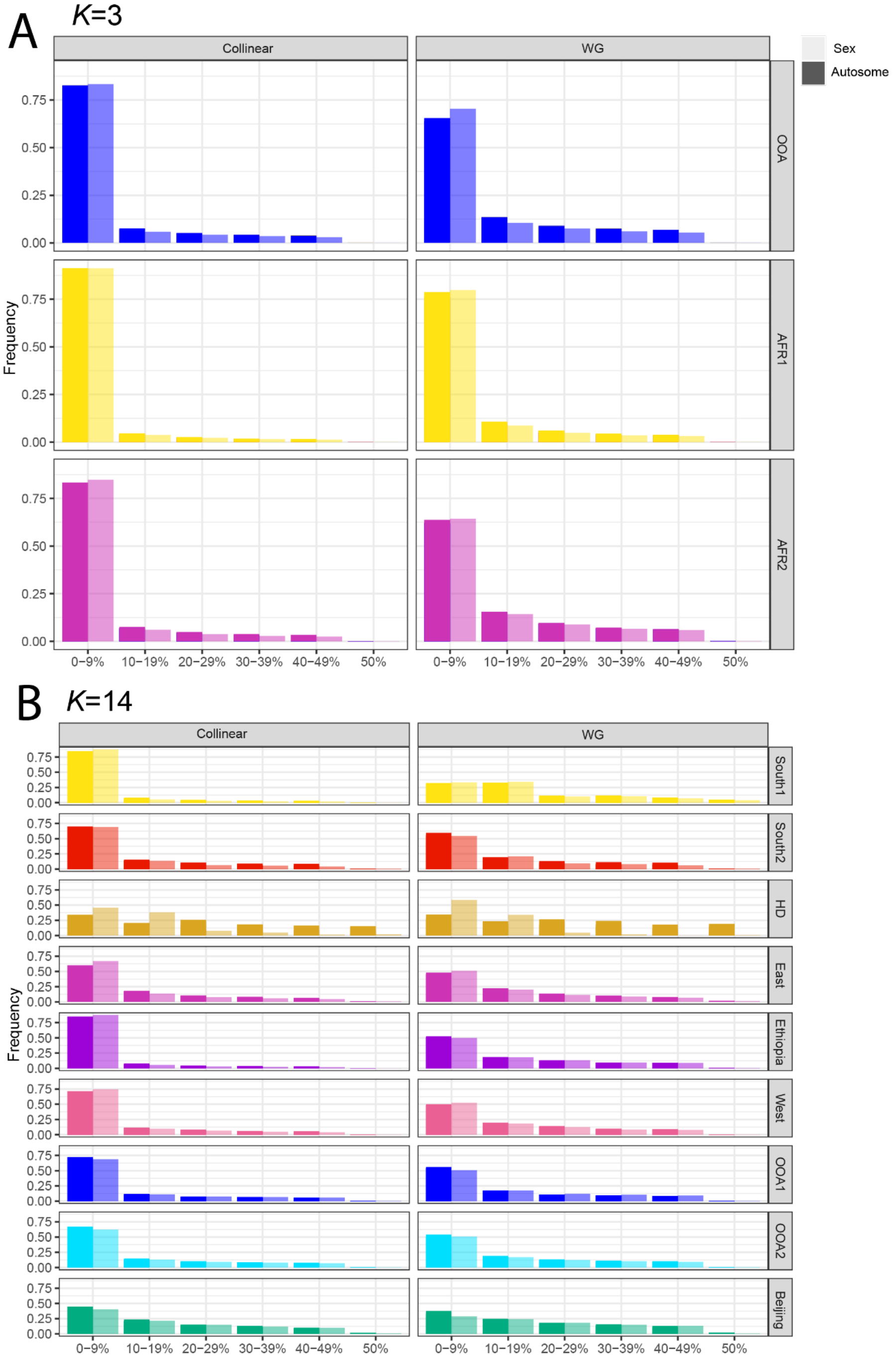

Figure S6: Folded Site-Frequency Spectrum (SFS). SFSs are shown for both autosomes and the *X* chromosome (dark versus translucent, respectively) for only collinear regions (left) and for the whole genome (WG; right) for both (A) *K=3* and (B) *K=14* ancestry designations.

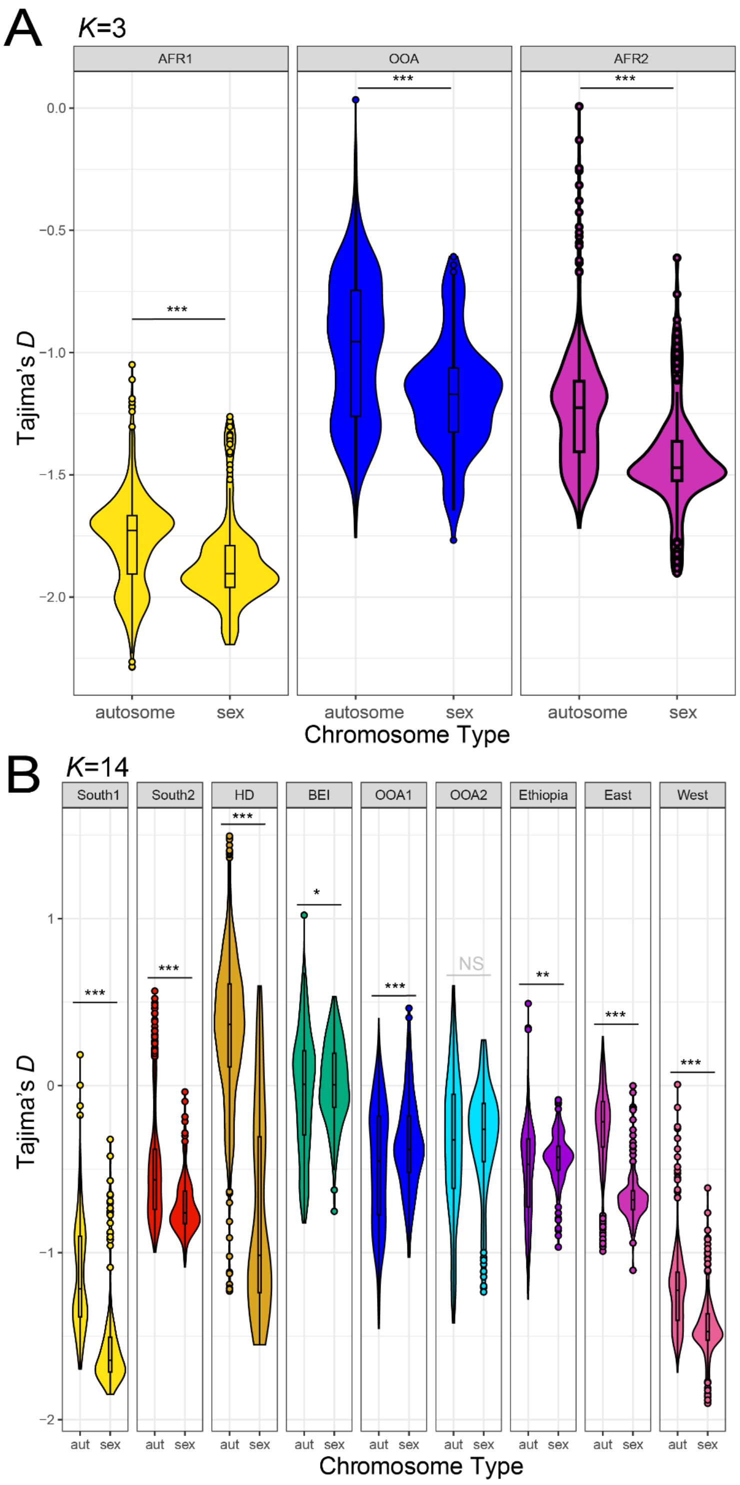

Figure S7: Tajima's D for each autosomal arm and the *X* chromosome for each ancestry, for (A) *K=3* and (B) *K*=14 ancestry designations.

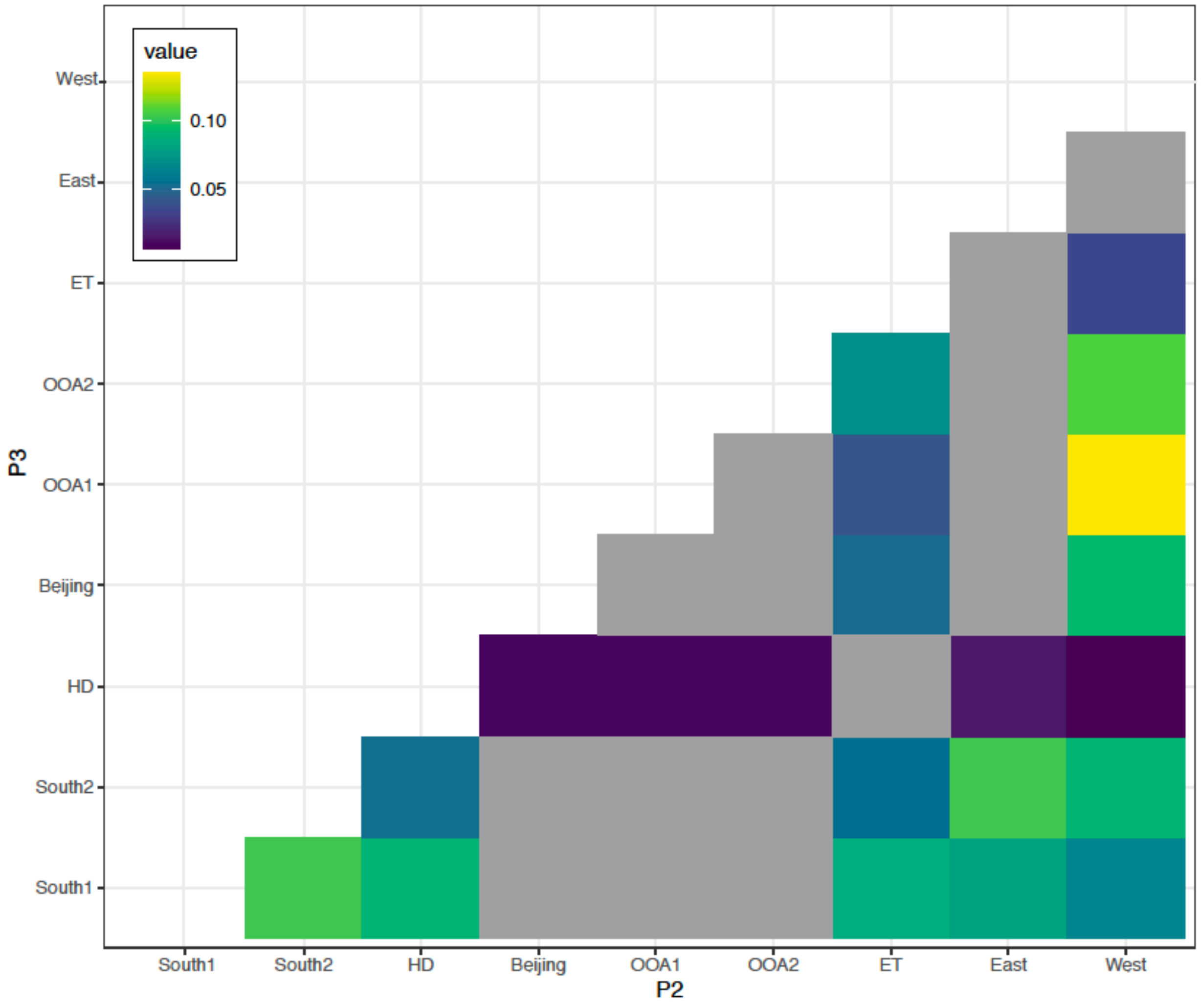

Figure S8: Average *f_G* between all P2-P3 combinations for our *K*=14 ancestries. Note that grey boxes denote either comparisons in which the ancestry on the X axis was never chosen as P2 (i.e. was always placed as P1), indicating no signal of introgression or comparisons that were not feasible to test with the inferred phylogeny (i.e. introgression among OOA lineages).

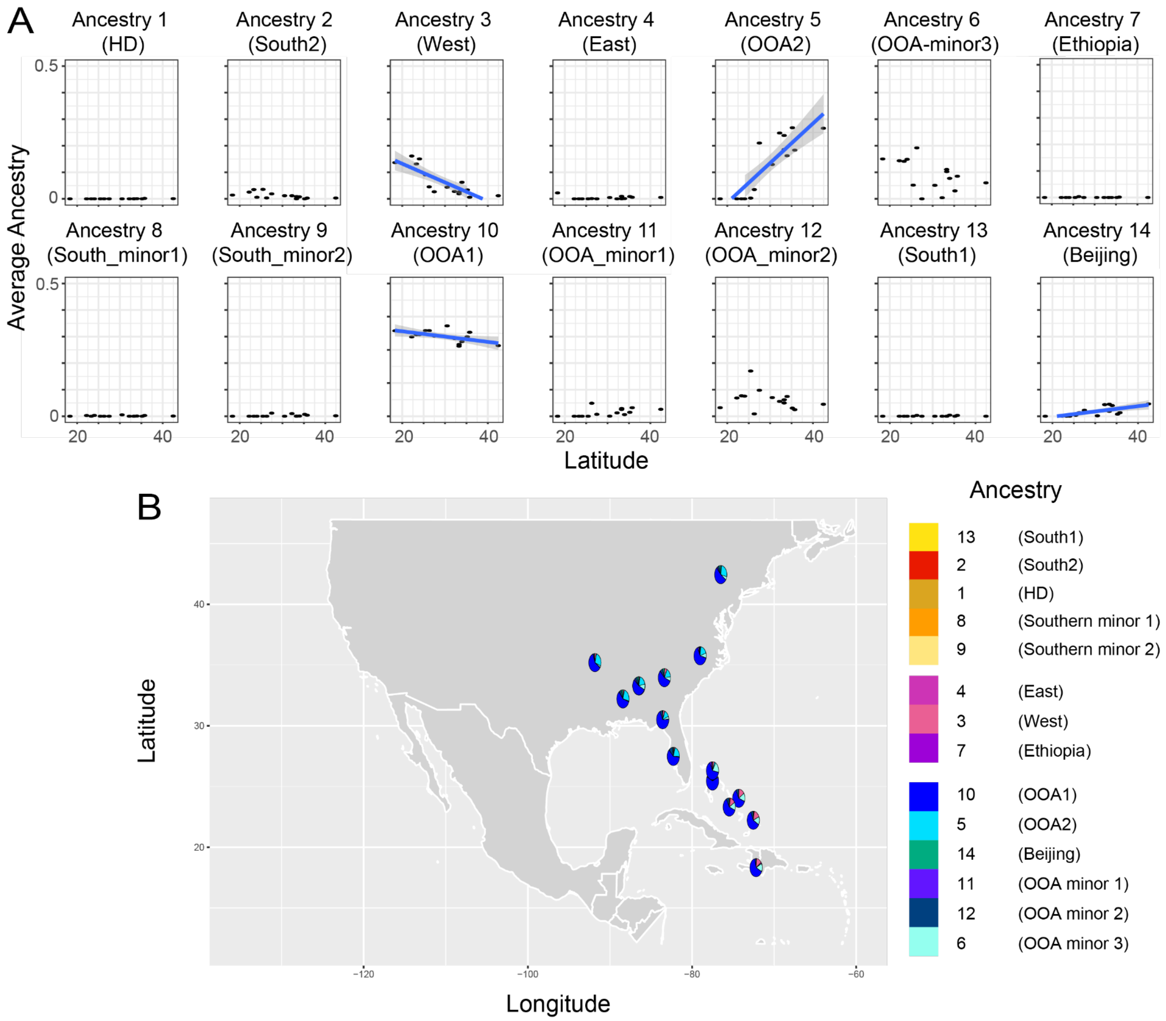

Figure S9: Latitudinal cline in West African ancestry for K=14. (A) All ancestries as a function of latitude in Caribbean and North American samples. Only 4 ancestries show a significant cline with latitude (West African, OOA1, OOA2, and Beijing). (B) Map of pie charts across the Caribbean and North America (excluding Winters, CA). Pie slices indicate the proportion of each ancestry type.

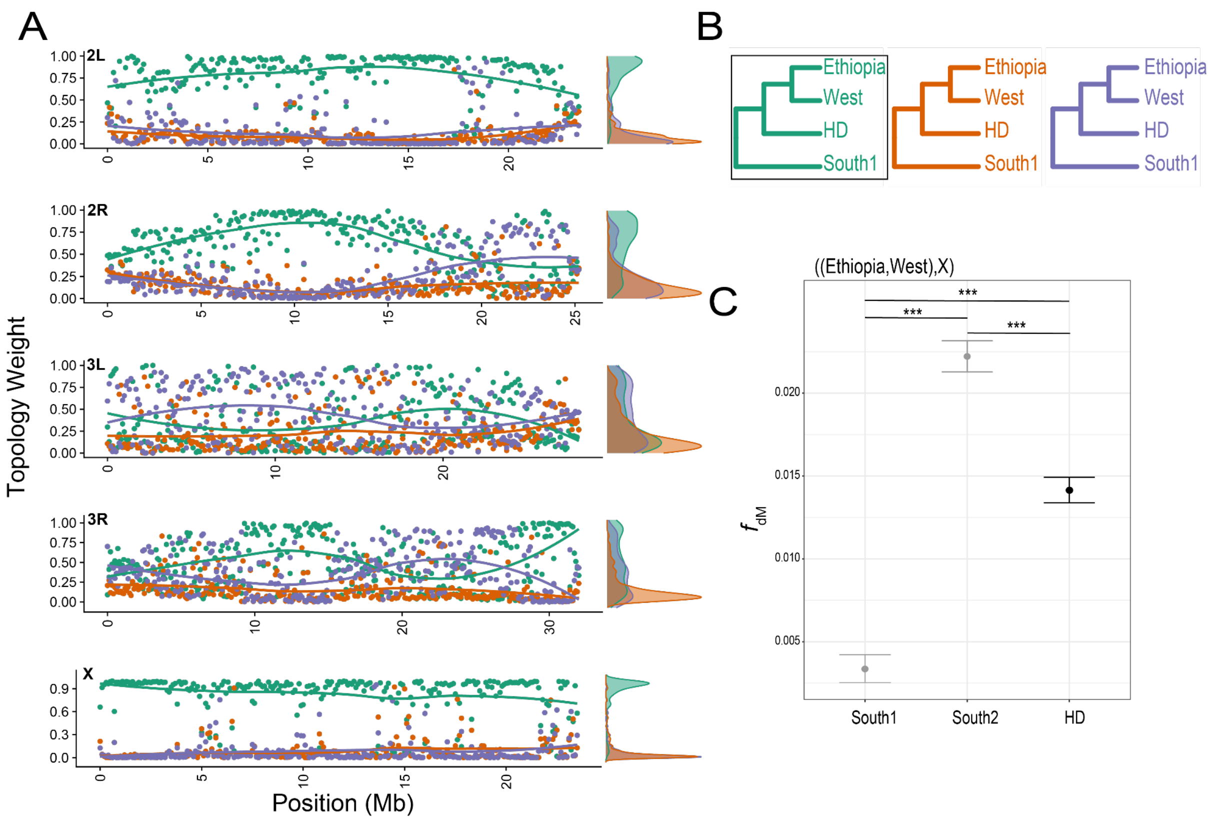

Figure S10: Landscape of introgression between West Africa and HD. (A) Weighted topologies from *twisst* based on 100kb windows across the genome. (A) The distribution of each topology across the chromosome/ chromosome arm. (B) Three topologies tested. A black box around the green phylogeny indicates the consensus tree based on ASTRAL. (C) Proportion of the genome inferred to have introgressed for each trio based on *D* statistics (f_{dM}). For each trio, we used the following topology (((Ethiopia,West),X),*simulans*), where X represents one of three Southern African ancestries. Points in grey (South1 and South2) were not found to exhibit significant introgression based on a genome-wide Patterson's *D.* Asterisks indicate significant differences between groups (p<0.0001).

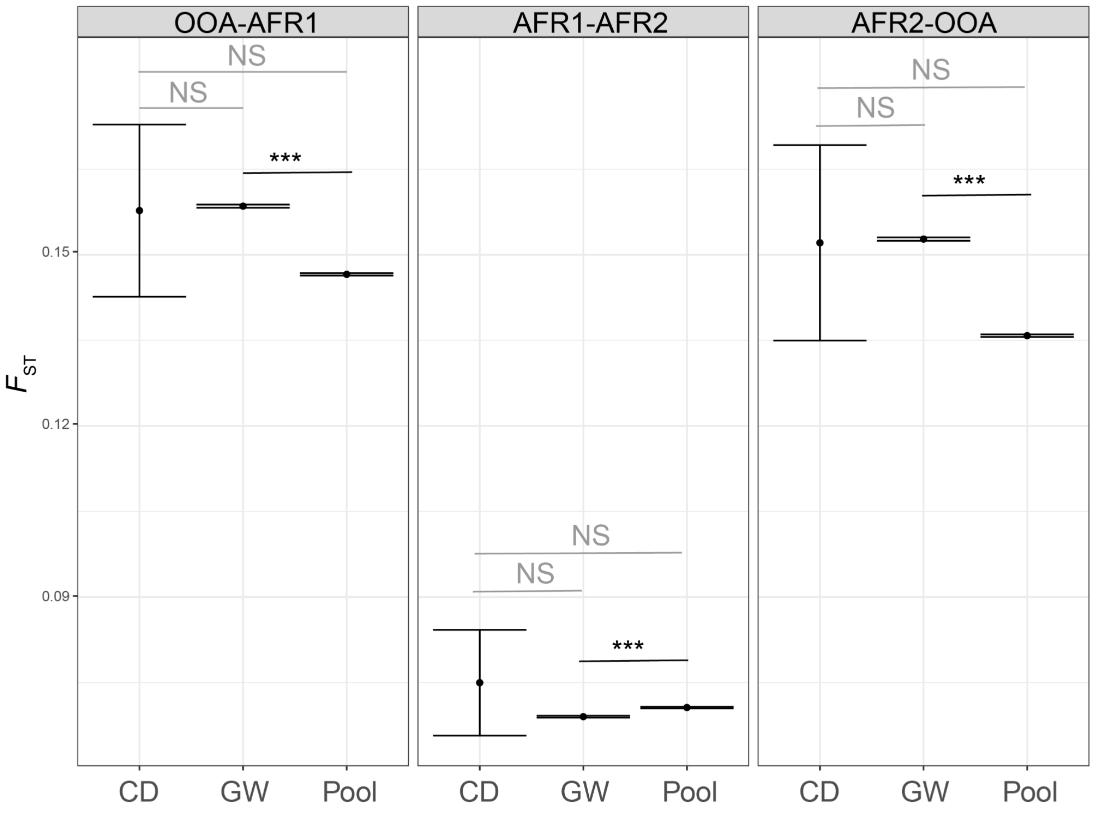

Figure S11: F_{ST} for all pairwise comparisons between ancestries when *K*=3 for two sets of potential incompatibility loci (CD= loci identified by (Corbett-Detig et al. 2013) and Pool= loci identified by (Pool 2015)) versus the genome-wide (GW) distribution of F_{ST}. We completed an ANOVA on a linear model to assess significant differences with the population pair, locus type, and their interaction as fixed effects. Pairwise differences were determined using estimated marginal means in the *emmens* package.

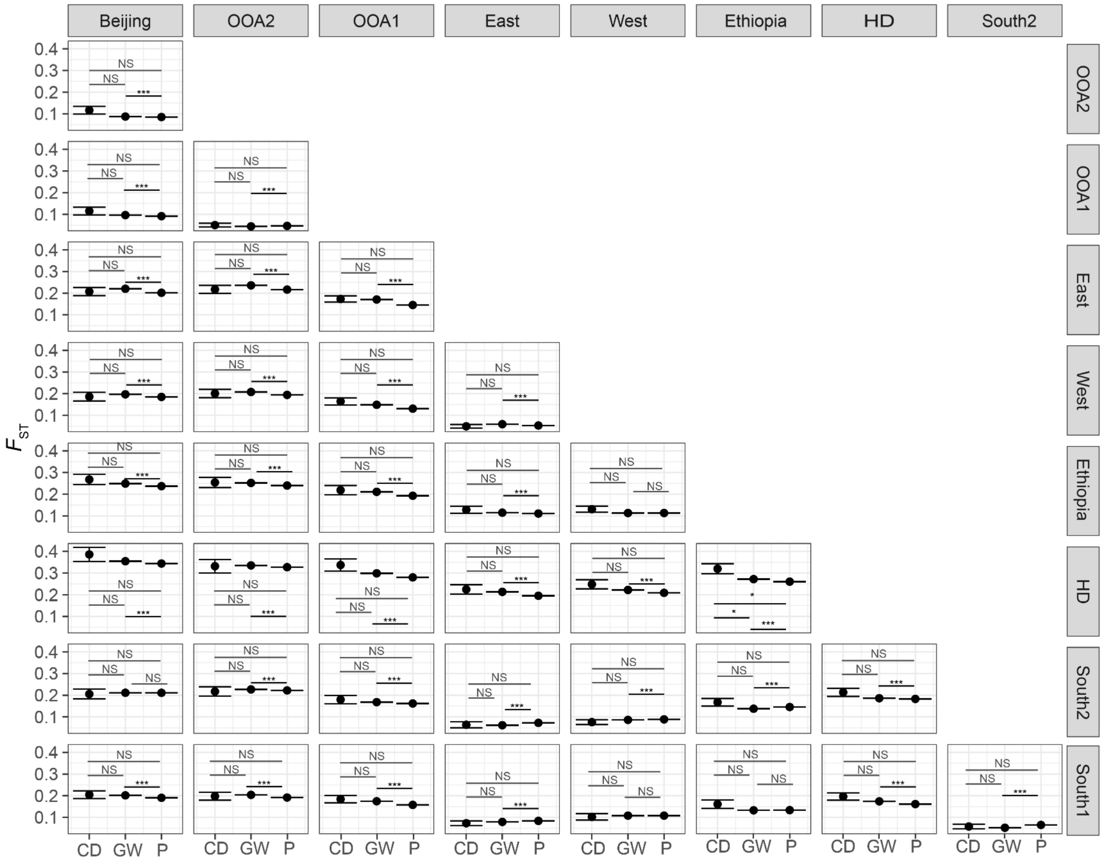

Figure S12: F_{ST} for all pairwise comparisons between ancestries when *K*=14 for two sets of potential incompatibility loci (CD= loci identified by (Corbett-Detig et al. 2013) and P= loci identified by (Pool 2015)) versus the genome-wide (GW) distribution of F_{ST}. Significance was determined by individual ANOVAs with pairwise *t*-tests.

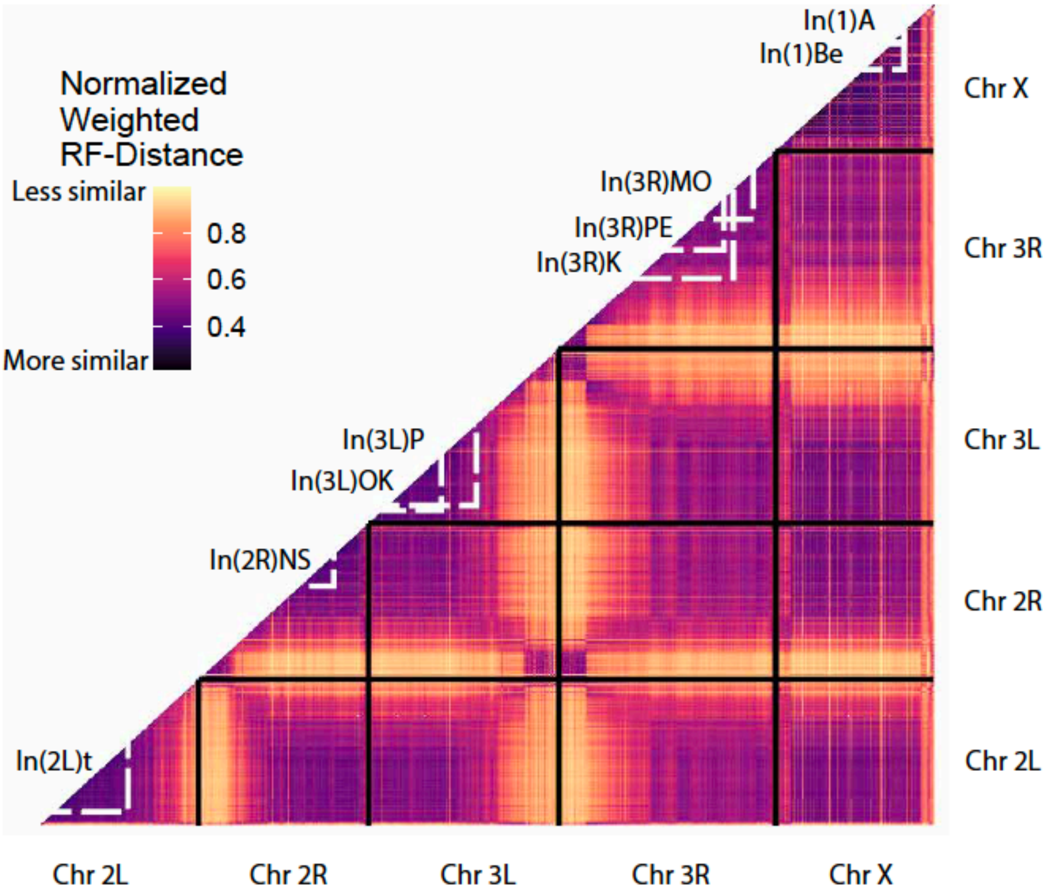

Fig. S13- Tree similarity across the genome. The heatmap shows normalized, weighted Robinson-Foulds distance between each 100kb tree across the genome. White dashed lines indicate the boundaries of the nine major inversion we exclude from the remainder of analyses. Note that only centromeres of the chromosomes show large differences from the remainder of the genome, but similarity to other centromeres.

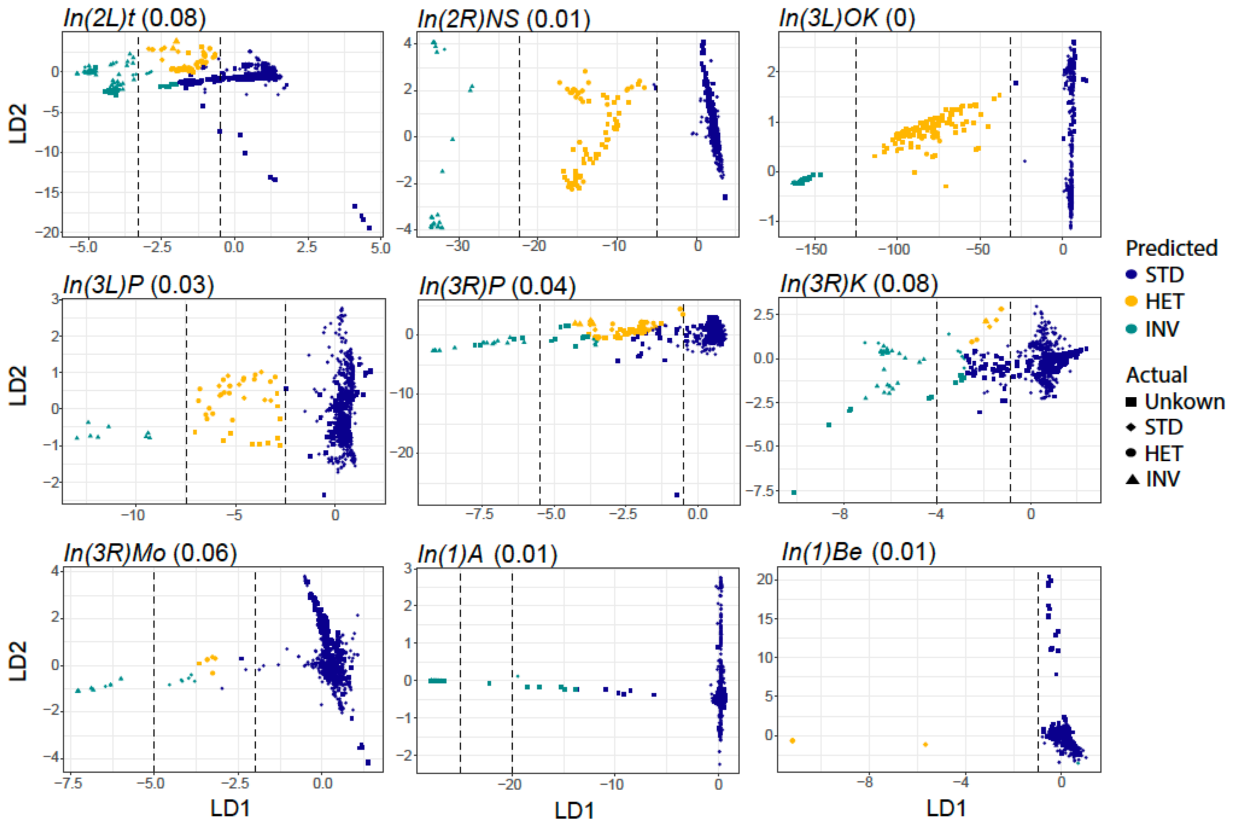

Fig. S14- Linear discriminant analyses based on ancestry for each of nine common inversions in *D. melanogaster.* Models were run on 75% of individuals with known karyotype, tested on the remaining 25% to obtain an error rate (denoted in parentheses above each plot). These models were then applied to the full dataset, including individuals with unknown karyotype. Dashed vertical lines represent the cutoffs for karyotype inference, wherein individuals to the far right were considered to be homozygous standard (STD) while individuals to the far left were considered homozygous inverted (INV). We refrained from calling heterozygous individuals for our new samples, except for three inversion regions where the three genotypes were clearly demarcated (*In*(2R)NS; *In*(3L)OK; *In*(3L)P).

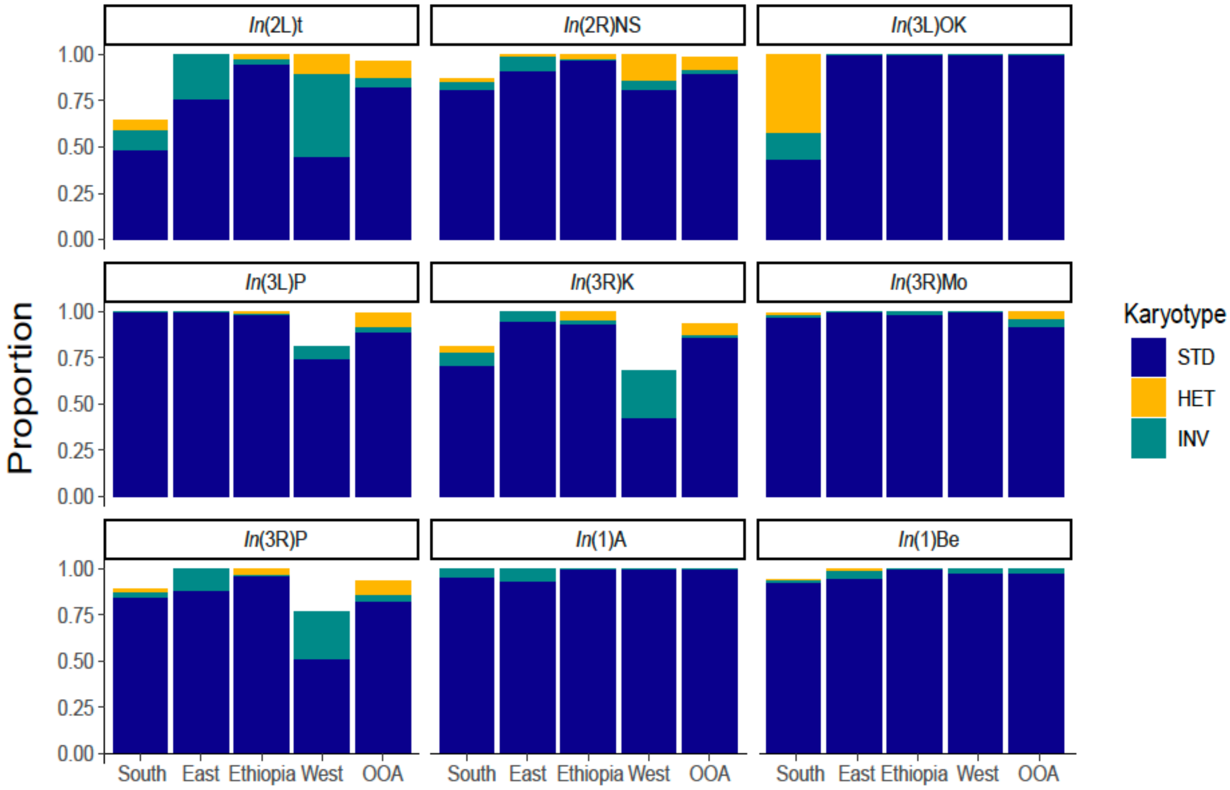

Fig. S15- Frequencies of three karyotypes for nine common chromosomal inversions across five major collection locales. These data are an amalgamation of previously determined inversion karyotypes (as in (Corbett-Detig and Hartl 2012; Lack et al. 2015)), as well as newly determined inversion karyotypes for 223 from Southern Africa. In some instances, bars do not sum to 1 as we applied conservative estimates, particularly in calling potentially heterozygous individuals (see Fig. S14 for thresholds).

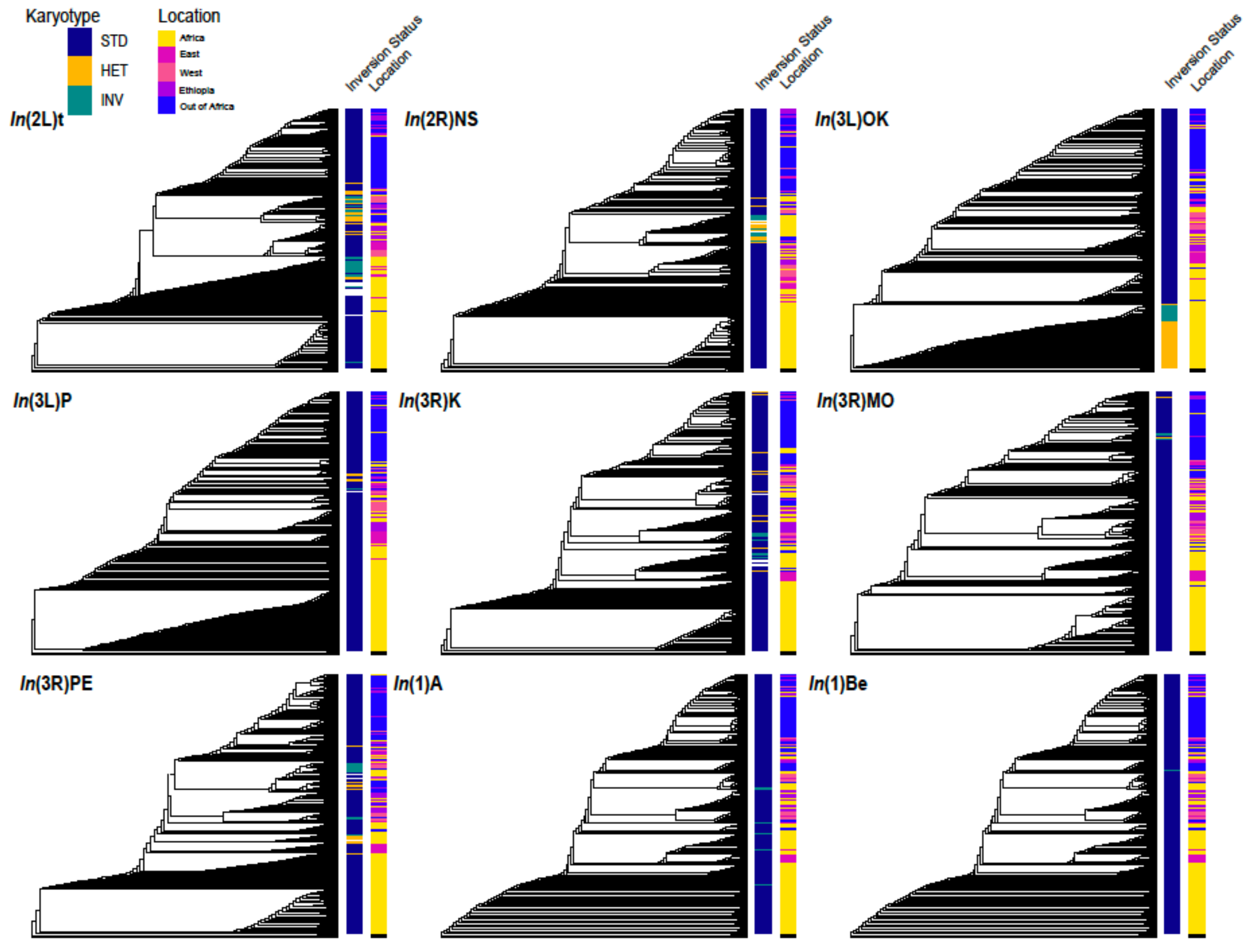

Fig. S16- Maximum likelihood ASTRAL phylogenies for 100kb windows for each of the nine common inversions in *D. melanogaster.* Color panels (from left to right) represent inversion karyotype and sampling location.

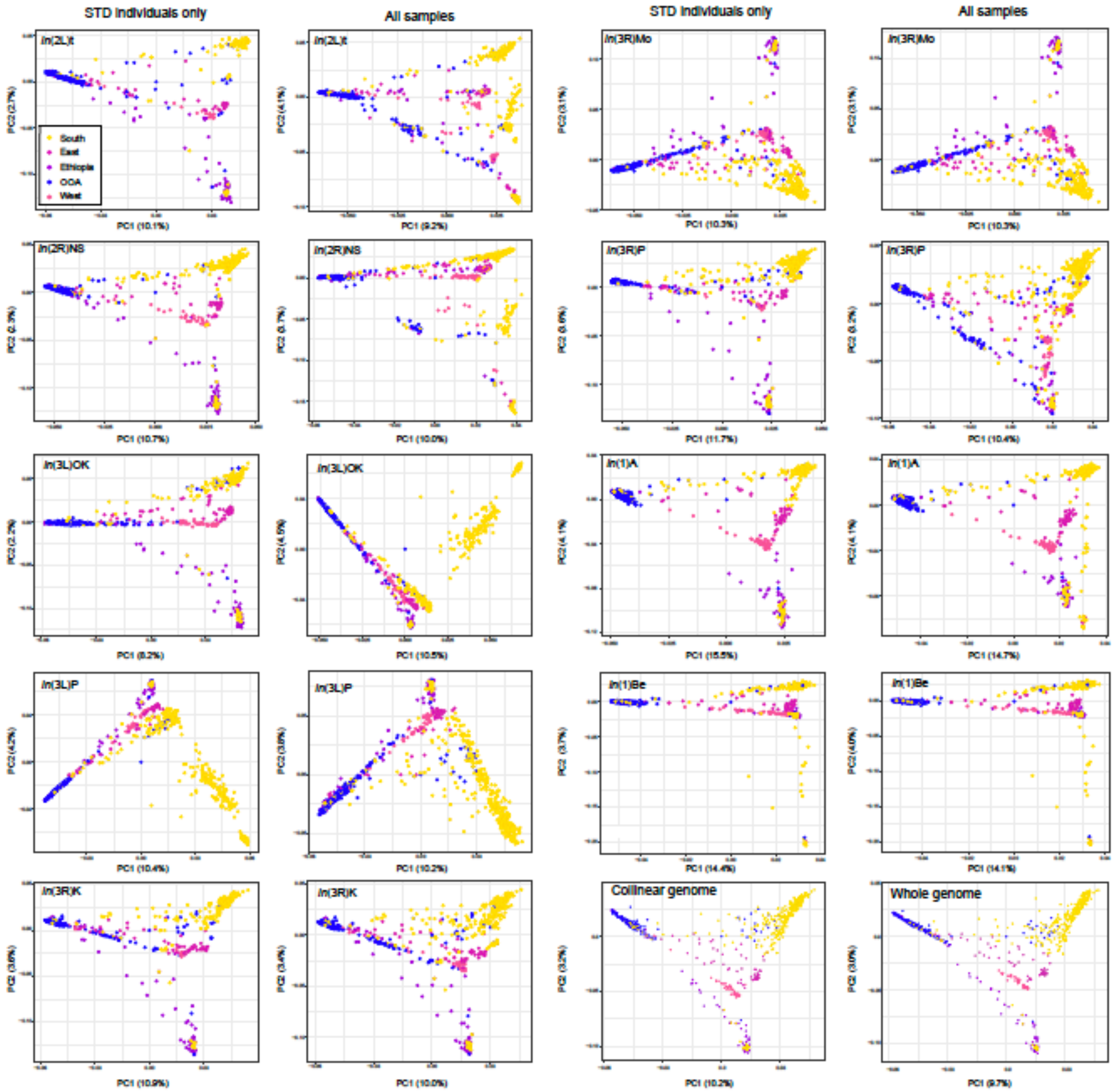

Fig. S17- PCAs based on nine common inversion regions in *D. melanogaster.* For each inversion, we present PCAs based on all individuals (of mixed karyotype; Right) and only individuals which are inferred to be homozygous for the standard arrangement (Left). Points are coloured based on five broad sampling locations. Bottom right two panels indicate the genome-wide (right) and collinear regions only. Percent of total variance explained by each PC is indicated in parentheses on each axes label.

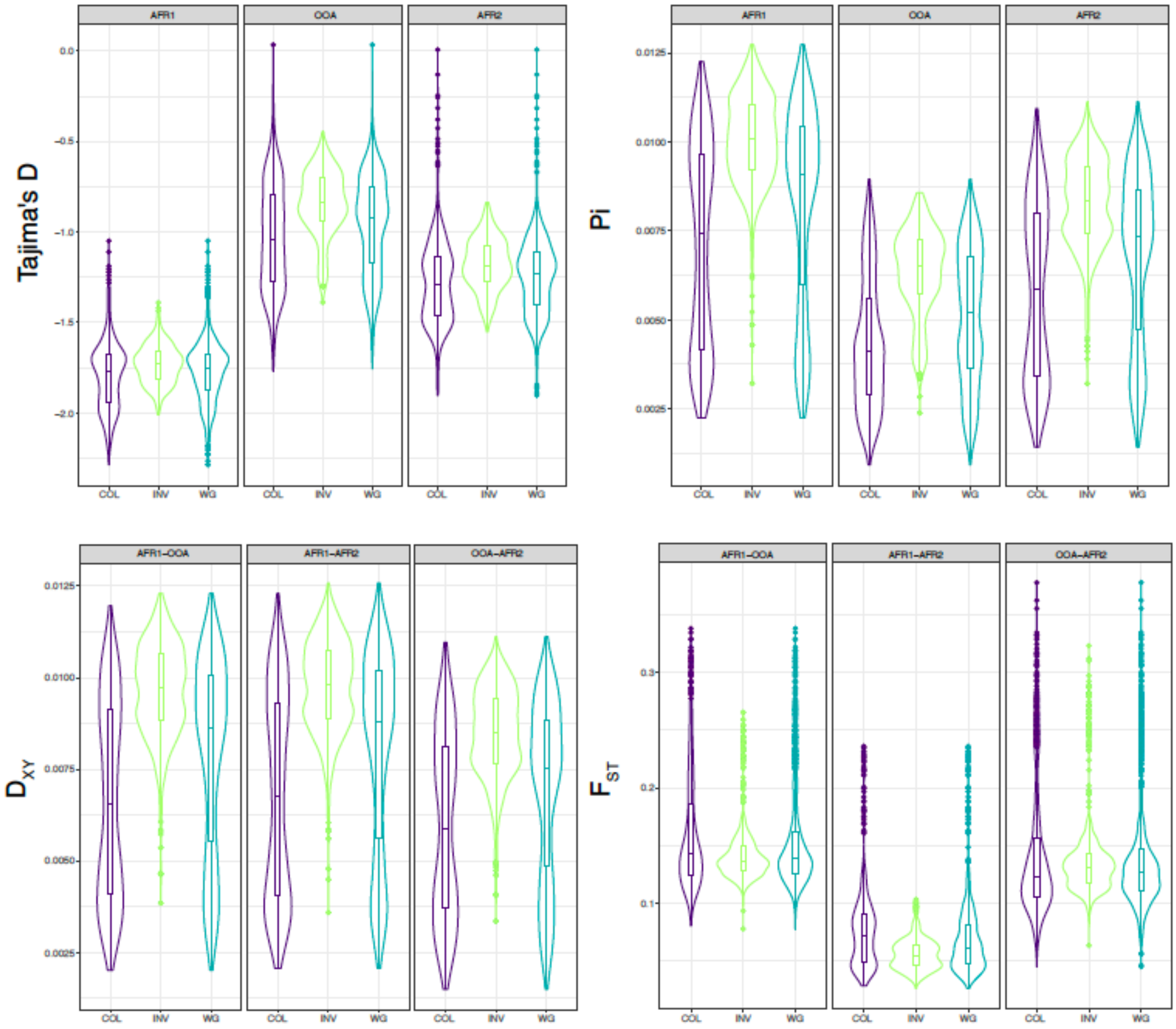

Figure S18: Population genetics statistics for three locus types: whole genome (WG), inversion regions only (INV), and collinear regions only (COL) for *K*=3 ancestries for all individuals (i.e. regardless of karyotype).

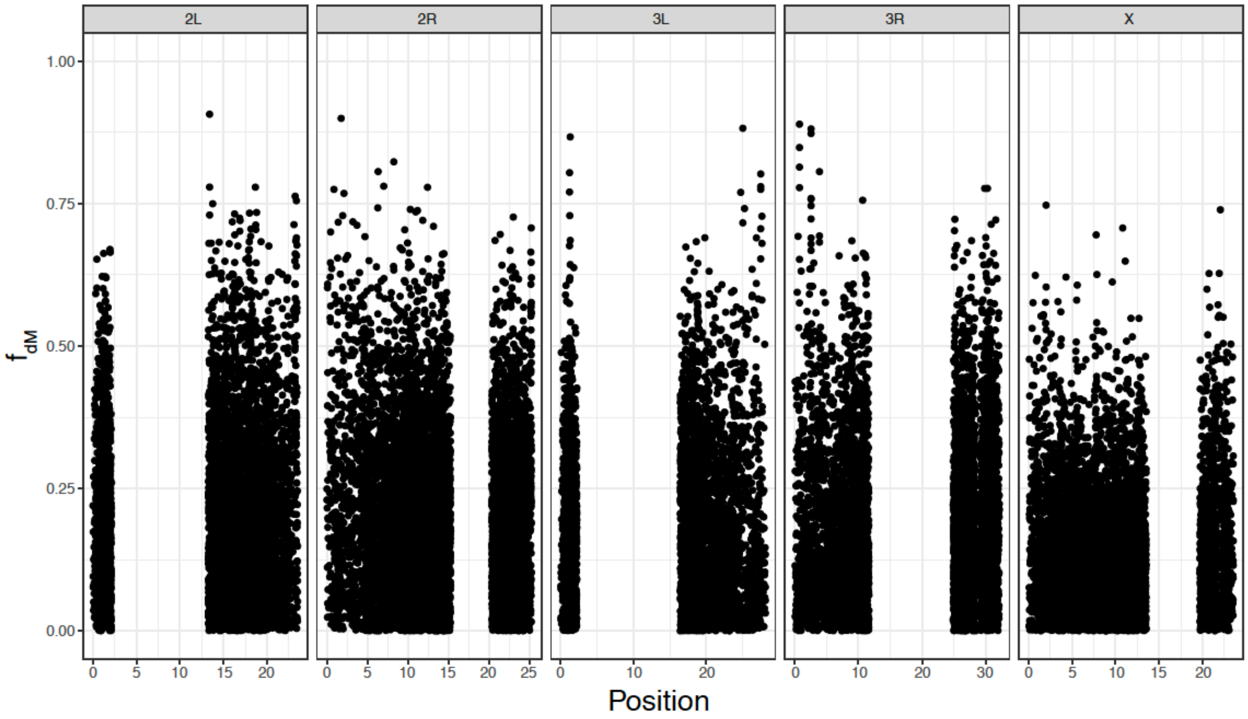

Figure S19: Landscape of introgression between West Africa and all OOA1 individuals, as depicted by f_{dM}. Only collinear regions of the genome are shown.

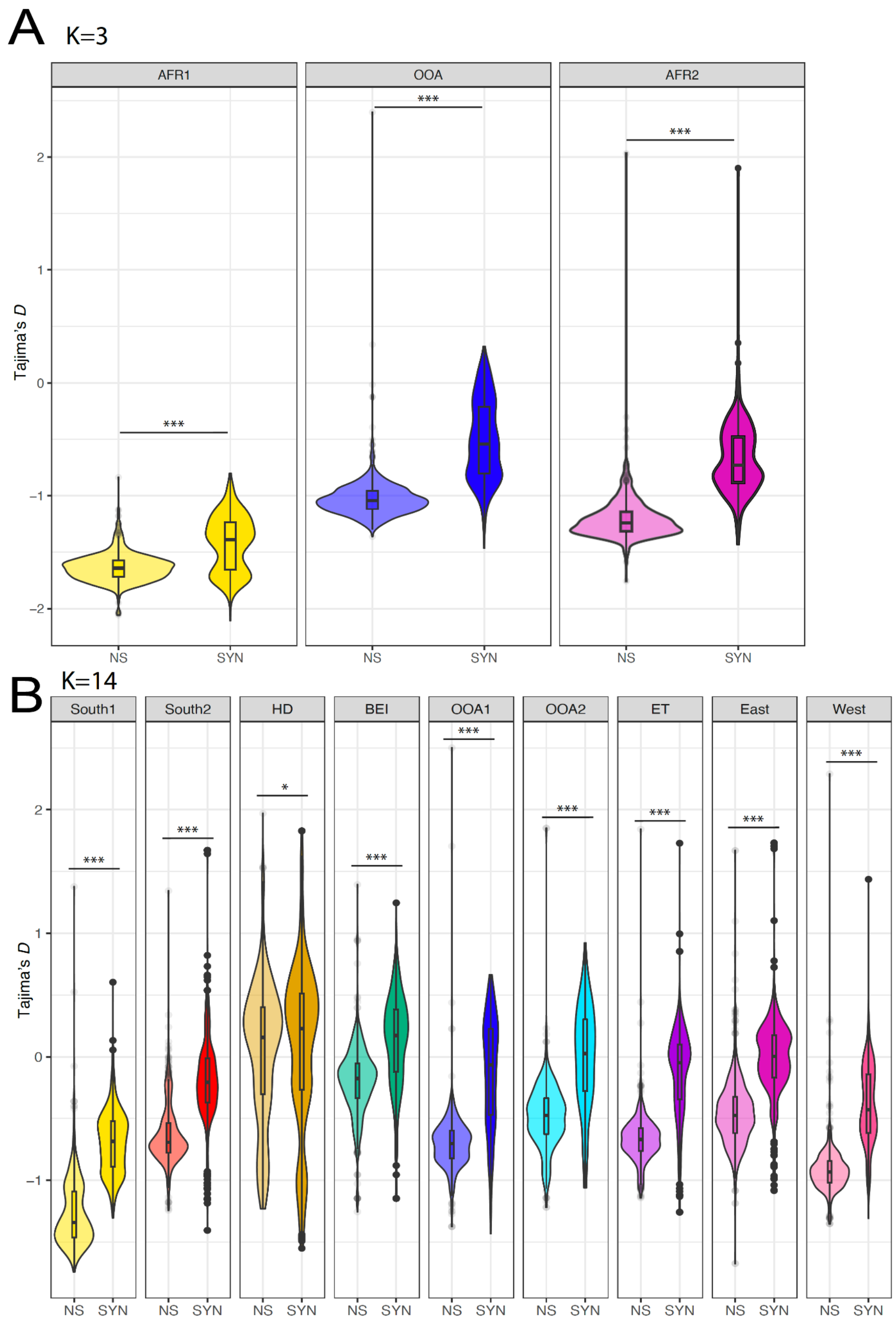

Figure S20: Tajima's *D* for genome-wide synonymous (SYN) and non-synonymous (NS) sites for each ancestry type for *K*=3 (A) and *K*=14 (B). Significant differences were determined using a linear model with Tajima's *D* as the response variable and ancestry, type of locus, and their interaction as the independent variables. Significance of fixed effects were assessed using an ANOVA with Type III Sum of Squares in the *car* package in R (Fox and Weisberg 2018), and specific comparisons were assessed using the *emmeans* package in R (Russell 2018)
